# Immune Profile and Clinical Outcome of Breakthrough Cases After Vaccination With an Inactivated SARS-CoV-2 Vaccine

**DOI:** 10.3389/fimmu.2021.742914

**Published:** 2021-09-29

**Authors:** Luisa F. Duarte, Nicolás M. S. Gálvez, Carolina Iturriaga, Felipe Melo-González, Jorge A. Soto, Bárbara M. Schultz, Marcela Urzúa, Liliana A. González, Yaneisi Vázquez, Mariana Ríos, Roslye V. Berríos-Rojas, Daniela Rivera-Pérez, Daniela Moreno-Tapia, Gaspar A. Pacheco, Omar P. Vallejos, Guillermo Hoppe-Elsholz, María S. Navarrete, Álvaro Rojas, Rodrigo A. Fasce, Jorge Fernández, Judith Mora, Eugenio Ramírez, Gang Zeng, Weining Meng, José V. González-Aramundiz, Pablo A. González, Katia Abarca, Susan M. Bueno, Alexis M. Kalergis

**Affiliations:** ^1^ Millennium Institute on Immunology and Immunotherapy, Santiago, Chile; ^2^ Departamento de Genética Molecular y Microbiología, Facultad de Ciencias Biológicas, Pontificia Universidad Católica de Chile, Santiago, Chile; ^3^ Departamento de Enfermedades Infecciosas e Inmunología Pediátricas, División de Pediatría, Escuela de Medicina, Pontificia Universidad Católica de Chile, Santiago, Chile; ^4^ Departamento de Enfermedades Infecciosas del Adulto, División de Medicina, Escuela de Medicina, Pontificia Universidad Católica de Chile, Santiago, Chile; ^5^ Departamento de Laboratorio Biomédico, Instituto de Salud Pública de Chile, Santiago, Chile; ^6^ Sinovac Biotech, Beijing, China; ^7^ Departamento de Farmacia, Facultad de Química y de Farmacia, Pontificia Universidad Católica de Chile, Santiago, Chile; ^8^ Departamento de Endocrinología, Facultad de Medicina, Escuela de Medicina, Pontificia Universidad Católica de Chile, Santiago, Chile

**Keywords:** CoronaVac, phase 3 clinical trial, SARS-CoV-2, COVID-19, vaccines, breakthrough cases

## Abstract

Constant efforts to prevent infections by severe acute respiratory syndrome coronavirus 2 (SARS*-*CoV*-*2*)* are actively carried out around the world. Several vaccines are currently approved for emergency use in the population, while ongoing studies continue to provide information on their safety and effectiveness. CoronaVac is an inactivated SARS-CoV-2 vaccine with a good safety and immunogenicity profile as seen in phase 1, 2, and 3 clinical trials around the world, with an effectiveness of 65.9% for symptomatic cases. Although vaccination reduces the risk of disease, infections can still occur during or after completion of the vaccination schedule (breakthrough cases). This report describes the clinical and immunological profile of vaccine breakthrough cases reported in a clinical trial in progress in Chile that is evaluating the safety, immunogenicity, and efficacy of two vaccination schedules of CoronaVac (clinicaltrials.gov NCT04651790). Out of the 2,263 fully vaccinated subjects, at end of June 2021, 45 have reported symptomatic SARS-CoV-2 infection 14 or more days after the second dose (1.99% of fully vaccinated subjects). Of the 45 breakthrough cases, 96% developed mild disease; one case developed a moderate disease; and one developed a severe disease and required mechanical ventilation. Both cases that developed moderate and severe disease were adults over 60 years old and presented comorbidities. The immune response before and after SARS-CoV-2 infection was analyzed in nine vaccine breakthrough cases, revealing that six of them exhibited circulating anti-S1-RBD IgG antibodies with neutralizing capacities after immunization, which showed a significant increase 2 and 4 weeks after symptoms onset. Two cases exhibited low circulating anti-S1-RBD IgG and almost non-existing neutralizing capacity after either vaccination or infection, although they developed a mild disease. An increase in the number of interferon-γ-secreting T cells specific for SARS-CoV-2 was detected 2 weeks after the second dose in seven cases and after symptoms onset. In conclusion, breakthrough cases were mostly mild and did not necessarily correlate with a lack of vaccine-induced immunity, suggesting that other factors, to be defined in future studies, could lead to symptomatic infection after vaccination with CoronaVac.

## Introduction

Severe acute respiratory syndrome coronavirus 2 (SARS-CoV-2) is a novel coronavirus first identified in China, in December of 2019, and is responsible of the current worldwide pandemic with nearly 4 million deaths reported at the beginning of July 2021 (1, 2). Coronavirus disease 2019 (COVID-19) is the result of infection caused by this virus, a disease that ranges from mild respiratory symptoms in over 80% of the population to severe illnesses requiring oxygen assistance and invasive ventilation, which usually leads to fatal or life-threatening outcomes (3).

Vaccine development has become the main hope for reducing COVID-19 cases and the severity of this disease (4). Several vaccines have been developed through different molecular approaches (i.e., viral mRNA, viral recombinant proteins, recombinant viral vectors, or inactivated whole virus), and up to date, the World Health Organization (WHO) has granted emergency approval for the use of 10 of them (5). Despite their differences, all these vaccines have reported a protective immune response against SARS-CoV-2 infections in clinical trials (6). Several studies have reported the production of antibodies with neutralizing capacities, along with broad cellular immune responses that helps in the clearance of the virus (6–10). However, breakthrough cases, defined as the detection of SARS-CoV-2 RNA in people ≥14 days after they completed the immunization schedule, have been reported (11, 12). These cases push the scientific community towards a further characterization and comprehension of the immune response elicited upon vaccination, in order to achieve enhanced protective responses in all the population.

CoronaVac is an inactivated SARS-CoV-2 vaccine that has shown to be 65.9%, 87.5%, 90.3%, and 86.3% effective in preventing COVID-19 symptoms, hospitalization, ICU admission, and COVID-19-related death, respectively, as recently reported in a cohort of almost 10.2 million individuals in Chile (13). It has been reported that immunization with CoronaVac elicits an immune response directed against several viral components, beyond the spike (S) protein, after the administration of two doses, as evidenced by detecting IgG antibodies against N protein and a substantial CD4^+^ T-cell response after *ex vivo* stimulation with a MegaPool (MP) of peptides covering the remainder “non-spike” SARS-CoV-2 proteome (7, 14, 15). Phase 3 clinical trials for this vaccine are being held in different countries around the globe (15, 16). Particularly in Chile, a clinical trial is undergoing to evaluate two different immunization schedules, with the second dose administered either 2 (0–14) or 4 (0–28) weeks after the first one (clinicaltrials.gov number: NCT04651790). Among 2,263 fully vaccinated volunteers, on June 25, 2021, a total of 45 COVID-19 cases (1.99%) have been reported occurring in the monitoring period (from 2 weeks after the second dose). Here, we report the clinical outcome and the immune response elicited by nine breakthrough cases detected among the 15 of the 450 volunteers enrolled in the immunogenicity branch of the phase 3 clinical trial, who already received both doses of CoronaVac. Evaluation of the humoral immune response considered the measurement of circulating anti-S1-RBD IgG antibodies and their neutralizing capacities as measured by two different techniques. Evaluation of the cellular immune response was performed through ELISPOT assays after *ex vivo* stimulation of peripheral blood mononuclear cells (PBMCs) with two sets of MP of peptides derived from the proteome of SARS-CoV-2 (17). A thorough understanding of the immune responses elicited after vaccination and as to how it correlates with the protection elicited after this and subsequent infections will provide valuable information that will improve the approaches currently being used to halt the COVID-19 pandemic and will also indicate whether an additional dose of currently approved vaccines is needed after a certain time span.

## Materials and Methods

### Study Design, Volunteers, and Randomization

The clinical trial (clinicaltrials.gov NCT04651790) was conducted in Chile at eight different sites and evaluated two immunization schedules in a 1:1 ratio. This trial was approved by each Institutional Ethical Committee and by the Chilean Public Health Institute (#24204/20) and conducted according to the current Tripartite Guidelines for Good Clinical Practices, the Declaration of Helsinki (18), and local regulations. Written informed consent was obtained from each participant. Volunteers included men and women aged ≥18, inoculated with two doses of 3 µg (600SU) of CoronaVac. One group received the second dose 2 weeks after the first dose (0–14 schedule), while a second group received the second dose 4 weeks after the first one (0–28 schedule). Exclusion criteria included, among others, history of confirmed symptomatic SARS-CoV-2 infection, pregnancy, allergy to vaccine components, and immunocompromised conditions. A complete list of inclusion and exclusion criteria has been published previously (15).

A total of 2,302 volunteers were enrolled by March 19, 2021, of whom 2,263 received both doses. A subgroup of 450 volunteers was selected to evaluate their immune response, receiving randomly CoronaVac either in a 0–14 or a 0–28 immunization schedule (1:1 ratio). Demographic information, comorbidities, nutritional status, immunization schedule, and dates of vaccination were obtained at enrollment and registered in the electronic case-report form (eCRF) for all volunteers. Nutritional status was determined using a gender and body mass index (BMI) (19).

### Breakthrough Case Follow-Up

Confirmed COVID-19 cases reported 14 days after the administration of the second dose of CoronaVac were identified following the protocol procedures for efficacy. Briefly, upon enrollment, participants were instructed to report through an electronic platform, e-mail, cell phone message, or telephone call, each time the definition for suspected positive case was met. A positive case was suspected if at least one of the following symptoms were present for over 2 days: fever or chills, coughing, shortness of breath or breathing difficulty, fatigue, muscle or body pain, headache, loss of smell or taste, sore throat, nasal congestion or runny nose, nausea or vomiting, and diarrhea. Upon the report, an evaluation visit was scheduled with a study physician, for 3 days after symptoms onset, to evaluate the presence of SARS-CoV-2 RNA by reverse-transcriptase quantitative PCR (RT-qPCR) in nasopharyngeal (NP) sample. If the sample was negative, and at least one symptom persisted, a second test was performed after 48 h. If a sample was positive, the clinical evolution of the case was closely monitored by the center personnel until its resolution. If hospitalization was required, information was obtained from relatives of the volunteer and from clinical reports.

Upon confirmation of positive cases, history of possible close contact with confirmed COVID-19 cases and the severity and duration of each signs and symptoms were registered. Severity was classified from grades 1 to 4, as published previously by the Food and Drug Administration (FDA) and the National Institutes of Health (NIH) (20, 21). Intensity of the disease was graded from score 1 to 9, as published previously by the WHO (22). The grading for severity criteria indicated in the protocol were either mild (symptomatic patients without viral pneumonia or hypoxia), moderate (clinical signs of pneumonia such as fever, coughing, shortness of breath, difficulty breathing but no signs of severe pneumonia, oxygen saturation ≥94% on room air), or severe {resting clinical signs indicative of severe clinical illness [respiratory rate (RR) ≥30/min; heart rate (HR) ≥125/min; oxygen saturation <94% at room air at sea level; PaO_2_/FiO_2_ <300 mm Hg], respiratory failure [requirement of high-flow oxygen, noninvasive ventilation, mechanical ventilation, or extracorporeal membrane oxygenation (ECMO)], evidence of shock [systolic blood pressure (SBP) <90 mmHg, diastolic blood pressure (DBP) <60 mmHg, or requirement of vasopressors], significant acute renal, hepatic, or neurological dysfunction, admission to ICU, or death}. All this information was recorded in both the clinical file of the participant and the eCRF.

### Procedures

To evaluate the immune response elicited upon immunization, peripheral blood samples were obtained for the isolation of serum and PBMCs. For volunteers from the immunogenicity branch, samples were collected before the first and the second dose and 2 and 4 weeks after the second dose. After COVID-19 confirmation by PCR, two additional peripheral blood samples were obtained about 2 and 4 weeks after symptoms onset (follow-up 1 and 2, respectively). Sera samples and PBMC were collected as previously reported (15) and stored at −80°C or in liquid nitrogen, respectively.

Circulating IgG antibodies specific against the RBD of the S1 protein of SARS-CoV-2 (S1-RBD) were measured using the COVID-19 Human Antibody Detection Kit (RayBio #IEQ-CoVS1RBD-IgG), following the instructions of the manufacturer. Sera samples were two-fold serially diluted, starting at a 200-fold dilution until a 6,400-fold dilution. The antibody titer was determined as the last fold dilution with an absorbance over the cut-off value. The cut-off value for each dilution was determined as 2.1 times the absorbance at 450 nm for a panel of 29 seronegative samples.

The neutralizing capacities of circulating antibodies were determined by two different techniques, i.e., through a surrogate virus neutralizing test (sVNT) and a conventional plaque-reduction neutralization test (cVNT). The sVNT were performed following the instructions of the manufacturer (BioHermes #COV-S41), and sera samples were 2-fold serially diluted starting at a 4-fold dilution until a 4,096-fold dilution. The percentage of inhibition was defined as follows: (OD_450 nm_ value of negative control − OD_450 nm_ value of sample)/(OD_450 nm_ value of negative control × 100), and titers were reported as the reciprocal of the highest serum dilution required to achieve 30% of inhibition. Samples exhibiting <30% inhibitory activity at the lowest dilution tested (1:4) were assigned a titer of 2. For the cVNT, sera samples were 2-fold serially diluted starting at a 4-fold dilution until a 512-fold dilution. Then, samples were incubated with a SARS-CoV-2 clinical isolate (33782CL-SARS-CoV-2 strain) for 1 h at 37°C. The mixtures were then added to Vero E6 cell monolayers (ATCC CRL-1586), and cytopathic effect (CPE) was evaluated 7 days after infection. Positive and negative controls were held for each assay. CPE was evaluated by direct visualization, and the titer of neutralizing antibodies was defined as the latest fold dilution exhibiting 100% of infection inhibition and absence of CPE. A titer of 2 was assigned for samples showing CPE at the lowest dilution tested (1:4).

The cellular immune response was evaluated through ELISPOT assays, as described previously, using the human interferon (IFN)-γ/IL-4 double-color ELISPOT (Immunospot) (15). Cells were cultured for 48 h in the presence of four different SARS-CoV-2-specific MPs (17). Two of these MPs are composed of 15-mer peptides derived from the S protein (MP-S) and the remaining proteins of the viral particle (MP-R). The other two MPs are composed of 9- to 11-mer peptides from the whole proteome of SARS-CoV-2 (CD8-A and CD8-B). Positives and negative controls were considered for each assay as reported previously (15, 17).

## Results

### Clinical Features of Breakthrough Cases

**Figure 1:**
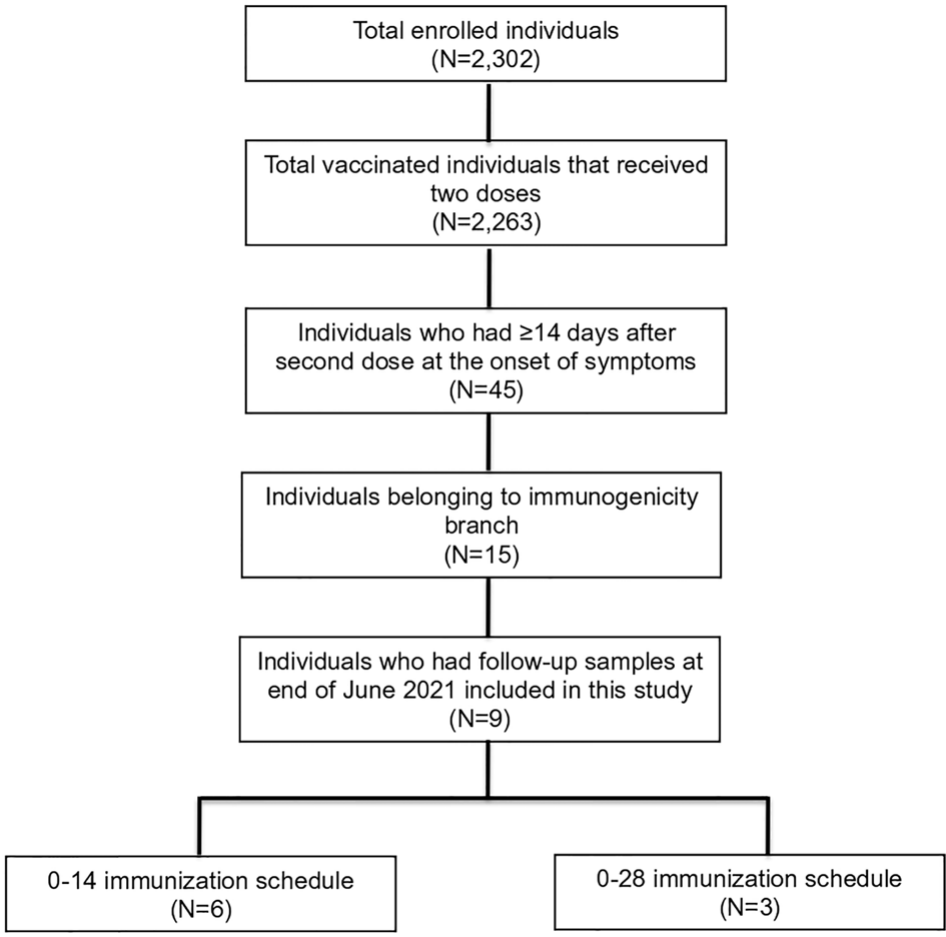
Enrolled volunteers and breakthrough cohort included in this study. Nine of the 2,302 vaccinated individuals belonging to the clinical trial conducted in Chile were included in this study after confirming COVID-19 disease by reverse-transcriptase polymerase chain-reaction (RT-qPCR) assay. They were selected from 45 individuals who displayed symptoms after ≥14 days from the administration of the second dose of the vaccine because they were enrolled in the immunogenicity branch and further had at least one follow-up sample after symptoms onset at the end of June of 2021.

From January 1 to June 25, 2021, 50 breakthrough cases were reported among the 2,263 vaccinated volunteers that had received two vaccine doses, of which 45 had over 14 days after the second dose (26 cases in the 0–14 schedule and 19 in the 0–28 schedule). Fifteen of these breakthrough cases were among the 450 volunteers in the immunogenicity branch. Eight of these had follow-up samples from days 14 and 30 after the start of symptoms of COVID-19, and one of them had a single follow-up sample taken 14 days after symptoms onset (Volunteer 1). All nine were Hispanic–Latin and were negative for the presence of circulating S- and N-SARS-CoV-2 IgG antibodies at recruitment. Six of them received the 0–14 immunization schedule and three the 0–28 immunization schedule (**Figure 1**). The demographic characteristics and relevant clinical history of cases are shown in **Table 1**.

**Table 1:** Demographic and clinical history of nine vaccine breakthrough cases.

Volunteer	Biological Sex*	Age	Nutritional Status	BMI	Co-morbidities
**1**	F	46	Normal	23.2	Migraine syndrome, allergic rhinitis
**2**	M	48	Overweight	28.9	Arterial hypertension, coronary heart
					disease, hypothyroidism
**3**	F	24	Overweight	25.3	Allergic rhinitis, penicillin allergy
**4**	M	62	Overweight	29.3	Hypothyroidism
**5**	F	32	Normal	23.9	Allergic rhinitis
**6**	F	33	Normal	20.5	Hypothyroidism
**7**	M	69	Overweight	28.0	Arterial hypertension, bicuspid aorta,
					atrial fibrillation, nephrolitiasis
**8**	F	28	Overweight	27.3	None
**9**	F	59	G2 Obesity	36.4	Insulin resistance

*Gray shading, female; no shading, male.

Intensity and severity of the disease were mild, with a score of 2 in seven out of the nine cases (Volunteers 1, 2, 3, 5, 6, 8, and 9), and the symptoms exhibited by them in decreasing frequency were nasal congestion (seven cases), sore throat (six), loss of smell (six), headache (five), coughing (four), loss of taste (four), runny nose (four), fatigue or myalgia (three), dyspnea (one), nausea (one), and diarrhea (one). None of the seven cases exhibited fever or vomiting. Accordingly, the duration of each symptoms was nasal congestion (1–13 days), sore throat (1–12), loss of smell (3–10), headache (5–13), cough (1–8), loss of taste (3–10), runny nose (2–13), fatigue (4–12), myalgia (1–21), dyspnea (12), nausea (4), and diarrhea (4–5). Most of the symptoms recorded were grade 1 or 2. The clinical outcome of the COVID-19 disease for each volunteer is indicated in **Table 2**.

**Table 2:** Clinical development of COVID-19 disease in the nine breakthrough cases described.

Volunteer*	Immunization schedule	Day of symptoms onset^	Possible close contact with COVID-19 case	Required Hospitalization	Highest clinical score
**1**	0–14	37	Yes	No	2
**2**	0–14	23	No	No	2
**3**	0–14	43	No	No	2
**4**	0–14	122	No	Yes	5
**5**	0–14	122	No	No	2
**6**	0–14	94	No	No	2
**7**	0–28	32	Yes	Yes	7
**8**	0–28	34	No	No	2
**9**	0–28	16	Yes	No	2

*Gray shading, female; no shading, male.

^^^Days after the administration of the second dose.

Two out of the nine breakthrough cases (Volunteers 4 and 7) reached a score over 2. The highest clinical score registered for Volunteers 4 was 5 (moderate), and for Volunteer 7 was 7 (severe). Volunteer 4 is a 62-year-old man, with a BMI of 29.3 (overweight) and is currently being treated for hypothyroidism (**Table 1**). The onset date was 122 days after the administration of the second dose (0–28 immunization schedule), and no close contact with a COVID-19-positive case was reported. The symptoms exhibited were fatigue, muscle pain, headache, nasal congestion, cough, and fever. After 6 days of disease development, Volunteer 4 was hospitalized due to persistent symptoms and the addition of shortness of breath to the list. A chest CT confirmed COVID-19 pneumonia. He was diagnosed with acute respiratory insufficiency and then received 4 L/min of oxygen by nasal cannula for 4 days. After this, he exhibited an overall improvement and recovery, with a total time of hospitalization of 8 days. Volunteer 7 is a 69-year-old man, with a BMI of 28.0 (overweight) and a history of arterial hypertension, bicuspid aorta, and atrial fibrillation. The onset date was 32 days after the administration of the second dose (0–28 immunization schedule), and close contact with a COVID-19-positive case was confirmed (his son). He presented respiratory symptoms and fever. Later, onset and persistence of malaise and fever, the onset of dyspnea, and the confirmation of COVID-19 pneumonia by a chest CT led to hospitalization. All the typical COVID-19 symptoms except nausea, vomiting, and diarrhea were reported after hospitalization. He received supplemental oxygen by nasal cannula and was transferred to ICU due to heart failure. He required mechanical ventilation for 6 days and eventually recovered, with a total time of hospitalization of 20 days.

Remarkably, as described below, two out of the nine breakthrough cases (Volunteers 2 and 6) exhibited a weak immune response upon immunization and infection. Volunteer 2 is a 48-year-old man, with a BMI of 28.9 (overweight) and a history of hypothyroidism, arterial hypertension, coronary heart disease (acute myocardial infarction on September 2020), fatty liver disease, and dyslipidemia under treatment. During his childhood, he was diagnosed with influenza-associated encephalitis (4 years old, hospitalized in ICU) and with uncomplicated diphtheria (6 years old). During his adulthood, he was diagnosed with a post-influenza pneumonia in 2000 and with a clinically suspected *Mycoplasma pneumonia* infection in 2018, both were treated with oral antibiotics. The symptoms onset was 26 days after the administration of the second dose (0–14 immunization schedule), and no contact with a COVID-19-positive case was reported. He presented fatigue, headache, nasal congestion, runny nose, coughing, and diarrhea. Volunteer 6 is a 33-year-old woman, with a BMI of 20.5 (eutrophic), and medical history of mononucleosis (2003), recurrent herpes simplex labialis (since 2003), hypothyroidism, and currently on oral contraceptive therapy. No contact with a COVID-19-positive case was reported, and the onset date was 94 days after the administration of the second dose (0–14 immunization schedule). She presented fatigue, muscular pain, loss of smell, loss of taste, sore throat, and nasal congestion.

Altogether, the immunization schedule, medical history, demographic characteristics, the symptoms onset day, reporting of close contact with COVID-19 confirmed cases, and the symptoms exhibited by all breakthrough cases are diverse, and an evident pattern of conditions leading to susceptibility towards SARS-CoV-2 infection is not observed.

### Humoral Immunity in Breakthrough Cases

**Figure 2:**
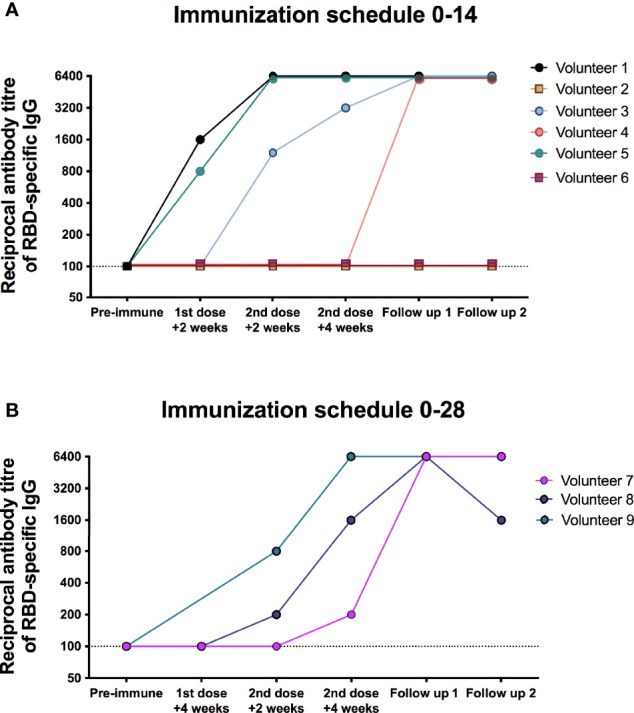
Circulating antibodies response elicited in the nine breakthrough cases measured as IgG specific against the S1-RBD of SARS-CoV-2. Specific IgG antibodies against the S1-RBD of SARS-CoV-2 were evaluated in nine breakthrough cases that received two doses of CoronaVac. The figure shows the antibody titer in the serum samples obtained before administration of the first dose (pre-immune), before administration of the second dose (1st dose + 2 weeks or 1st dose + 4 weeks), 2 and 4 weeks after the second dose, and 2 and 4 weeks after the disease onset and a confirmed PCR result for SARS-CoV-2 (follow-up 1 and 2, respectively) and a confirmed PCR result for SARS-CoV-2. **(A)** shows the six volunteers enrolled in the 0–14 immunization schedule, and **(B)** shows the three volunteers enrolled in the 0–28 immunization schedule.

To evaluate the humoral immune response elicited by the nine breakthrough cases, circulating IgG antibodies specific against the S1-RBD of SARS-CoV-2 were evaluated as indicated in *Materials and Methods*. As shown in **Figure 2** (and individually for each volunteer in **Supplementary Figure S1**), three out of the six cases from the 0-14 immunization schedule (Volunteers 1, 3, and 5) exhibited detectable levels of IgG antibodies specific against the S1-RBD at 4 weeks after the administration of the second dose (**Figure 2A** and **Supplementary Figures S1A, C, E**). This was also found for all three subjects in the 0–28 immunization schedule, although Volunteer 7 showed a weak response (**Figure 2B** and **Supplementary Figures S1G**–**I**). Circulating antibodies specific against S1-RBD also increased drastically 2 and 4 weeks after disease onset for all volunteers, except for Volunteers 2 and 6, that exhibited no changes in their antibodies profile throughout the time points evaluated.

**Figure 3:**
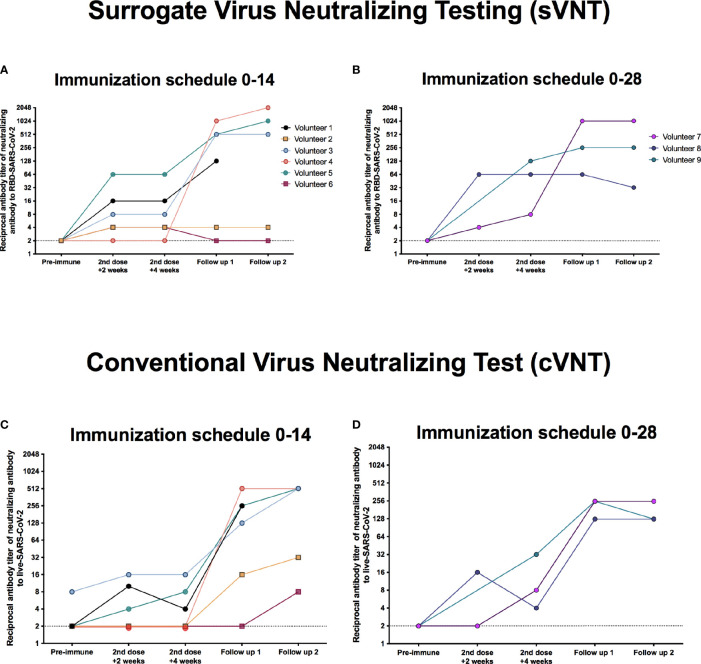
Circulating antibodies exhibit varying neutralizing capacities in the nine breakthrough cases. Neutralizing antibodies were evaluated before administration of the first dose (pre-immune), 2 and 4 weeks after the second dose, and 2 and 4 weeks after the disease onset (follow-up 1 and 2, respectively). Two different techniques were used, a surrogate virus neutralization test (sVNT) based on the perturbation of the hACE2-spike protein–protein interaction mediated by antibodies, and a conventional virus neutralization test (cVNT) evaluating plaque and CPE reduction. **(A)** Neutralizing antibody titers detected by using the sVNT in six volunteers enrolled in the 0–14 immunization schedule. **(B)** Neutralizing antibody titers detected by using the sVNT in three volunteers enrolled in the 0–28 immunization schedule. **(C)** Neutralizing antibody titers detected by using the cVNT in six volunteers enrolled in the 0–14 immunization schedule. **(D)** Neutralizing antibody titers detected by using the cVNT in three volunteers enrolled in the 0–28 immunization schedule.

The neutralizing capacities of the circulating antibodies measured in these nine breakthrough cases were also evaluated by two different techniques, as indicated in *Materials and Methods*. As evaluated by sVNT, five out of six cases in the 0–14 immunization schedule exhibited detectable levels of neutralizing antibodies 4 weeks after the administration of the second dose (**Figure 3A** and **Supplementary Figures S2A**–**F**). As expected, Volunteers 2 and 6 exhibited a very weak neutralizing capacity at this time point evaluated. However, upon evaluation by cVNT, only three volunteers in the 0–14 immunization schedule (Volunteers 1, 3, and 5) showed detectable neutralizing response (**Figure 3C**), which is in line with the results obtained for IgG antibodies specific against the S1-RBD (**Figure 2A**). Notably, no neutralizing capacities were detected for the antibodies of Volunteer 4 (who displayed a moderate disease development) 2 or 4 weeks after the second dose, for both sVNT and cVNT (**Figures 3A, C**). All three cases in the 0–28 immunization schedule had detectable levels of neutralizing antibodies, by both sVNT and cVNT, 2 and 4 weeks after the administration of the second dose (**Figures 3B, D**). Noteworthy, Volunteer 7 (who developed severe symptoms) exhibited a very weak neutralizing capacity at these time points evaluated. As also seen for the circulating IgG antibodies specific against the S1-RBD, the neutralizing capacities of most volunteers increased drastically 2 and 4 weeks after the onset of disease symptoms, even for Volunteer 4, who exhibited no response after vaccination (**Figures 3A**–**D**).

### IFN-γ Releasing by T Cells in Breakthrough Cases

**Figure 4:**
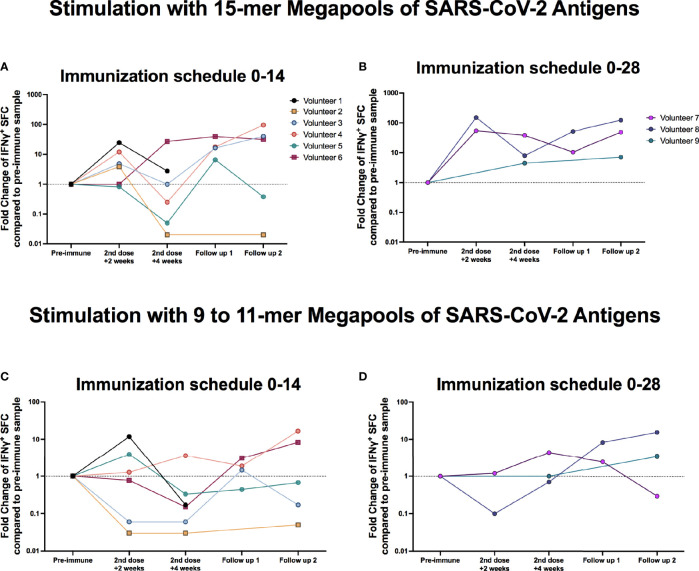
The IFN-γ production by T cells from breakthrough cases after stimulation with MegaPools of SARS-CoV-2 peptides is heterogeneous. PBMCs from the nine breakthrough cases were obtained before administration of the first dose (pre-immune), 2 and 4 weeks after the second dose, and 2 and 4 weeks after the disease onset (follow-up 1 and 2, respectively) and evaluated by ELISPOT assays. Cells were stimulated for 48 h with two MPs containing several peptides from SARS-CoV-2 to induce the secretion IFN-γ by T cells. The number of spots-forming cells (SFCs) was evaluated. Data are shown as the fold increase regarding to the preimmune value for SFCs. **(A)** Fold change of IFN-γ^+^ SFCs after stimulation with MPs containing 15-mer peptides from SARS-CoV-2 of six volunteers enrolled at the 0–14 immunization schedule. **(B)** Fold change of IFN-γ^+^ SFCs after stimulation with MPs containing 15-mer peptides from SARS-CoV-2 of three volunteers enrolled at the 0–28 immunization schedule. **(C)** Fold change of IFN-γ^+^ SFCs after stimulation with MPs containing 9- to 11-mer peptides from SARS-CoV-2 of six volunteers enrolled at the 0–14 immunization schedule. **(D)** Fold change of IFN-γ^+^ SFCs after stimulation with MPs containing 9- to 11-mer peptides from SARS-CoV-2 of three volunteers enrolled at the 0–28 immunization schedule.

To evaluate the cellular immune response elicited in these nine breakthrough cases, ELISPOT assays were performed as seen on **Figure 4** and **Supplementary Figure S3**. The number of spot-forming cells (SFC) positive for IFN-γ upon stimulation with MPs of peptides derived from SARS-CoV-2 were measured, as described in *Materials and Methods*. For most volunteers, upon stimulations with MPs containing 15-mer peptides (MP-S and MP-non-spike), SFC values measured in samples obtained 2 weeks after the administration of the second dose exhibited at least a two-fold increase as compared to those obtained before the administration of the first dose (**Figure 4A** for the 0–14 immunization schedule and **Figure 4B** for the 0–28 immunization schedule). Interestingly, Volunteer 6 showed no remarkable changes in the SFC values up to 4 weeks after the second dose, similar to that observed for Volunteer 9. SFC values increased for all volunteers (except Volunteer 2) 2 or 4 weeks after disease onset. Overall, SFC values obtained were higher when stimulating with MPs containing 15-mer peptides compared to those obtained when stimulating with MPs containing 9- to11-mer peptides (MP-CD8A and B) for both immunization schedules (**Figures 4A, C** for the 0–14 immunization schedule and **Figures 4B, D** for the 0–28 immunization schedule). Remarkably, Volunteer 6 displayed a good cellular response both after vaccination and infection, despite exhibiting a poor humoral response. The variation in SFC values for each volunteer after stimulation of MP-S and MP-non-spike and MP-CD8A and B is shown in **Supplementary Figure S3** and **Supplementary Tables 1**, **2**.

Overall, the results suggest that the cellular immune response elicited after either vaccination or infection in these nine breakthrough cases does not necessarily correlate with protection against SARS-CoV-2.

### Immune Responses of Vaccine Breakthrough Cases as Compared to a Control Cohort

For the purpose of better understanding whether the immune response elicited after vaccination in breakthrough cases was an exclusive feature and a determining factor in the susceptibility to the further infection, we compared the humoral and cellular-mediated immune response of breakthrough cases with the response observed in a control group of individuals vaccinated with similar characteristics to the breakthrough population, but without manifestation of clinical symptoms related to COVID-19. Control cohort consisted of 18 subjects who received two doses of CoronaVac on similar dates to the breakthrough cases and shared demographic characteristics as detailed in **Supplementary Table 3**.

**Figure 5:**
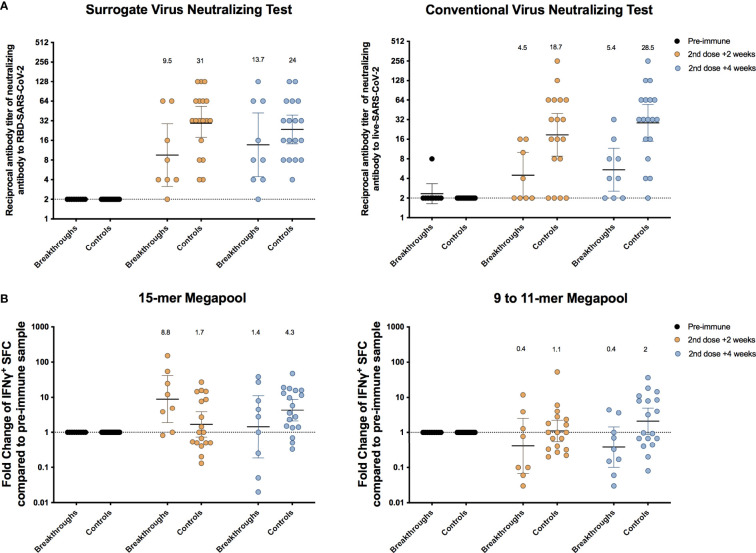
Humoral and cellular immune responses of breakthrough cases as compared to a control cohort. A control cohort of 18 subjects who received two doses of the CoronaVac was selected by matching with breakthrough cases (2:1 ratio) according to the biological sex, range of age, and schedule of vaccination. **(A)** Titers of antibodies able to inhibit RBD-SARS-CoV-2 interaction with ACE2 receptor or surrogate virus neutralizing test (sVNT, left) and titers of neutralizing antibodies against infective SARS-CoV-2 or conventional virus neutralizing test (cVNT, right) detected in the breakthrough and control cohort. Serum samples were obtained before administration of the first dose (preimmune), 2 and 4 weeks after the second dose. The numbers above the spots indicate GMT, and error bars show the 95% CI of the GMT. **(B)** Fold change of IFN-γ^+^ SFCs after stimulation of PBMCs with MPs containing 15-mer peptides (left) and 9- to 11-mer MPs (right) from SARS-CoV-2 proteome in the breakthrough and control cohort. PBMCs were obtained before administration of the first dose (preimmune), 2 and 4 weeks after the second dose. The numbers above the spots indicate geometric mean of the fold increase regarding to the preimmune sample, and error bars show the 95% CI. GMT, geometric mean titer; PBMCs, peripheral blood mononuclear cells; MPs, megapools.

As observed in **Figure 5A**, breakthrough cases show neutralizing antibodies titers about two-fold lower than the control group for sVNT, with geometric mean titers (GMTs) of 9.5 (95% CI, 3.1–28.7) vs. 31 (95% CI, 17.8–53.2) and 13.7 (95% CI, 4.5–42.2) vs. 24 (95% CI, 14.2–38.9), 2 and 4 weeks after the second dose, respectively. In a similar way, the GMTs in the breakthrough group were approximately four-fold lower than those obtained by the control cohort for cVNT, 4.5 (95% CI, 2–10) vs. 18.7 (95% CI, 8.8–39.6) and 5.4 (95% CI, 2.5–11.6) vs. 28.5 (95% CI, 15–54.6), 2 and 4 weeks after the second dose, respectively. Importantly, these trends were sustained when titers of neutralizing antibodies from six additional breakthrough cases, which had data available for samples after vaccination, were added to the analysis (**Supplementary Figure S4**).

Conversely, we observed a better cellular response after stimulation with 15-mer MPs in the breakthrough cases than the control group at 2 weeks after the second dose administration, which decreased at 4 weeks after the second dose to lower levels than the control group. Regarding the 9- to 11-mer MPs stimulating (mainly CD8^+^ T cells), a greater response was observed in the control group but only in approximately 50% of the individuals at 4 weeks after the second dose (**Figure 5B**).

In summary, these results show that detection of low levels of neutralizing antibodies after vaccination could be related to symptomatic infection; however, unknown underlying conditions must be affecting this susceptibility because low titers were also observed in some individuals belonging to the control group and high titers in the breakthrough group.

## Discussion

The use of different vaccines approved for emergency use due to the rapid spread of SARS-CoV2 has been key in stopping the uncontrolled progression of deaths worldwide. However, it has been reported that people with comorbidities can develop a more severe disease upon infection with SARS-CoV-2 (23). In this line, the efficacy of these vaccines can be impaired by the existence of previously described diseases or pathologies (24). In addition, the severity of the disease can be even more pronounced in the elderly, as they exhibit higher dysfunction in their immune system as compared to young people (25).

In this clinical trial, a total of 2,263 volunteers were vaccinated with two doses in two different immunization schedules. Out of all these volunteers, a total of 450 were part of the immunogenicity profile evaluation group. Here, we report the clinical outcome and immune response elicited by nine volunteers from the immunogenicity branch that were infected with SARS-CoV-2 and developed mild, moderate, or severe cases of COVID-19. Our results showed that the humoral and cellular immune response elicited by breakthrough CoronaVac cases was heterogeneous, and at least in these nine individuals, a correlate of infection was not evident. Yet, older people have a greater susceptibility to develop severe diseases as compared to younger people.

Of these nine volunteers, six exhibited some degree of overweight, and only one volunteer did not have any comorbidity. Two volunteers developed diseases that required hospitalization. Volunteer 7, a 69-year-old man, reported four comorbidities and required mechanical ventilation. Volunteer 4, a 62-year-old man, reported two comorbidities and required supplemental oxygen. Remarkably, in line with the results shown here, various publications have suggested that men are more prone to severe cases of COVID-19 and deaths than women, and this is even more pronounced in older populations (26, 27). Overweight and obesity are one of the most common comorbidities reported in critical patients suffering severe cases of COVID-19 (28). Furthermore, it has been reported that patients with elevated BMI exhibit more severe infection than patients with normal BMI (a high BMI is usually defined as ≥25) (29). This point is critical, as Volunteers 4 and 7 had a BMI of 28·0 and 29·3, respectively.

The particular bad evolution presented by Volunteer 7 could be partially explained by his underlying hypertension, and its corresponding treatment, which could induce an overexpression of angiotensin-converting enzyme 2 (ACE2), the receptor used by SARS-CoV-2 to infect target cells (30). Cardiac diseases have also been strongly associated with an increase in the susceptibility of SARS-CoV2 infection, the severity of COVID-19, and the susceptibility to death, as drugs used to control these illness may result in the overexpression of ACE2 in the heart (31, 32).

The hypothyroidism reported for Volunteer 4 has been related to increased susceptibility to severe COVID-19, as it affects the expression of ACE2 (33). Hypothyroidism may also be a factor predisposing the development of cardiac diseases, which increase the susceptibility of SARS-CoV-2 infection (33). As Volunteer 4 reported fewer comorbidities than Volunteer 7 (and therefore probably less risk factors to acquire SARS-CoV-2 and develop more severe COVID-19), a better prognosis would have been expected, which is in line with the information reported here.

Two volunteers out of the nine breakthrough cases did not exhibit a detectable immune response after immunization with CoronaVac. Volunteers 2 and 6 were younger than 60 years old and were of different sex. Volunteer 2 was a male with overweight (BMI, 28.9) and several comorbidities such as hypothyroidism arterial hypertension, coronary heart disease, fatty liver disease and dyslipidemia. He also reported a medical history of several infectious diseases in his childhood and adulthood. The circulating antibodies of this volunteer showed a poor neutralizing capacity, and there was a practically null induction of IFN-γ-secreting T cells after both vaccination doses and even after infection with SARS-CoV-2. Despite this, the degree of the disease reported in this subject was mild, and he did not require hospitalization or oxygen assistance, but it is possible that innate immunity also played a key role in the protection of this individual or that antigen-specific adaptative immune responses were not detected, since they could be restricted to mucosae or lungs (34, 35). Volunteer 6 was a female with normal weight and comorbidities such as hypothyroidism. The circulating antibodies of this volunteer showed a poor neutralizing capacity, but unlike Volunteer 2, she developed a robust cellular response after 4 weeks of vaccination which was also increased after disease onset. Although the number of breakthrough cases between both immunization schedules are not balanced, it is important to note that Volunteer 2 and 6 were vaccinated in the 0–14 schedule, which has been reported to induce a lower seroconversion rate and GMTs than the 0–28 schedule (36). Interestingly, both volunteers had hypothyroidism as a common comorbidity, which could affect the induction of the immune response and produce a dysregulation of the immune system (37). In this line, more in-depth studies are required to understand which factors could be involved in these poor responses and how they could impact in the future with the appearance of new circulating variants of SARS-CoV-2.

Limitations of this study include the sample size and the focus on self-reporting to identify breakthrough vaccine infections. Asymptomatic infections were not discarded and could therefore be missed in the cohort chosen as control, which in turn may cause a misinterpretation of the results regarding the comparison with the immune response elicited by the breakthrough cases. Therefore, our conclusions are directed toward the correlation of protection to suffer a symptomatic infection. On the other hand, only in Volunteer 4 the Gamma variant was identified by molecular analysis, and these data remained unknown for the rest of the breakthrough cases analyzed (Volunteer 6, 7, and 9). Hence, we lack evidence to determine whether the frequency of breakthrough vaccine cases is related to community transmission of a particular variant, which, in the case of Chile, has been dominated by the SARS-CoV-2 variants Gamma and Lambda in recent months (38).

Despite the low number of breakthrough cases included in this report, our results provide a clear and extensive clinical and immune description of mild, moderate, or severe infections exhibited after full vaccination with CoronaVac and support previous evidence that a poor induction of neutralizing antibodies after vaccination could be correlated to a decrease in the vaccine efficacy (39–41). Furthermore, data presented here provide valuable information over the potential role that play the underlying comorbidities on the vaccine effectiveness, which could impair the ability of an individual to activate a robust immune response after vaccination, and increase the risk of severe COVID-19 in elderly people. This information could be helpful and timely support the need of a booster dose in susceptible individuals with underlying conditions after a specific time to increase its protection.

Although the information presented here must be interpreted with caution because the sample size is small to generalize, some strengths of our study are worth noting, such as the serial testing after vaccination and infection and the measurement of T-cell responses in addition to humoral response. Previous reports have been focused on viral sequence information or antibodies detection on samples obtained after the onset of symptoms (11, 12, 39, 42, 43). This new information could be the interest to the scientific community and health authorities due to the urgent need to understand the individual variables that predispose to breakthrough infections and further find a correlate of protection that has not been established to date for SARS-CoV-2 infections; yet, some studies suggest that the level of neutralizing antibody titers is highly predictive of immune protection (40, 41). In this regard, our serial sample data reveal some key features: first, older volunteers 4 and 7 who presented moderate and severe illness, respectively, displayed the weakest humoral response after vaccination, but conversely, they showed the highest level of neutralizing antibodies titers after infection. Notably, susceptibility to infection was irrespective of the immunization schedule, as one of them belonged to the 0–14 immunization schedule and the other one to the 0–28. Second, younger people could not be able to elicit a good humoral immune response after vaccination or subsequent infection, as shown by volunteers 2 and 6. These observations could be explained, at least in part, by the presence of some comorbidities in these individuals and highlighted the importance of combining clinical information along with immunogenicity and efficacy studies. Finally, individuals with evidence of neutralizing antibodies elicited by vaccination can also become sick, but this is more likely to course with a mild infection (Volunteers 1, 3, 5, 8, and 9). Importantly, we observed that the level of neutralizing antibodies in this breakthrough cohort was lower than that in controls without a confirmed SARS-CoV-2 infection, but it remains to be determined what titers of antibodies are needed to prevent infection.

On the other hand, since the approval for the emergency use of CoronoVac, the WHO has encouraged addressing the current knowledge gap about the vaccine efficacy through assessment and reporting of breakthrough infections by using neutralization and T-cell immunity assays (44). To our knowledge, this is the first time that cellular-mediated response is reported for breakthrough vaccine cases. Our results showed that breakthrough cases had a good T-cell response elicited after vaccination but that was more associated to CD4^+^ than CD8^+^ T cells. A similar response was observed after infection, with only a volunteer not responding (Volunteer 2). It is important to note that not only cellular response to spike protein was evidenced but also to others viral antigens, as shown after stimulation with the megapool R (**Supplementary Figure 3**). However, it is not clear whether both humoral and T-cells responses are needed for protection, and further studies are needed to address that issue.

In summary, vaccination with CoronaVac is effective, and vaccine breakthrough cases showed mainly mild symptoms of COVID-19, even in those who did not exhibit a potent humoral immune response, which could be possibly associated with different risk factors as overweight and other comorbidities that could impair the immune response induced upon immunization. While additional data have become available to draw more robust conclusions, this evidence and information could be useful to the countries that actually have implemented CoronaVac in their vaccination campaigns and to guide future vaccination program policies.

## Data Availability Statement

The original contributions presented in the study are included in the article/**Supplementary Material**. Further inquiries can be directed to the corresponding authors.

## Ethics Statement

The studies involving human participants were reviewed and approved by Comité Ético Científico de Ciencias de la Salud UC, Pontificia Universidad Católica de Chile. The patients/participants provided their written informed consent to participate in this study.

## Author Contributions

Conceptualization: AK, KA, SB, PG, JG-A, GZ, and WM. Visualization: AK, KA, SB, PG, JG-A, GZ, and WM. Methodology: RF and JM. Investigation: LD, NG, CI, FM-G, JS, BS, MU, RB-R, LG, GH-E, DM-T, GAP, MR, DR-P, OV, YV, MN, and ÁR. Funding acquisition: AK. Project administration: AK, KA, SB, and PG. Supervision: AK, KA, SB, and PG. Writing—original draft: LD, NG, JS, CI, and MU. Writing—review and editing: AK, KA, SB, and PG. All authors contributed to the article and approved the submitted version.

## Funding

This work was supported by The Ministry of Health, Government of Chile and the Confederation of Production and Commerce (CPC), Chile, through the funding of the CoronaVac03CL Study. The Millennium Institute on Immunology and Immunotherapy, ANID—Millennium Science Initiative Program ICN09_016 (former P09/016-F), supports SB, KA, PG, and AK. The Innovation Fund for Competitiveness FIC-R 2017 (BIP Code: 30488811-0) supports SB, PG, and AK. SINOVAC contributed to this study with the investigational vaccine and experimental reagents.

## Conflict of Interest

ZG and MW are SINOVAC employees and contributed to the conceptualization of the study (clinical protocol and eCRF design) and did not participate in the analysis or interpretation of the data presented in the manuscript.

The remaining authors declare that the research was conducted in the absence of any commercial or financial relationships that could be construed as a potential conflict of interest.

## Publisher’s Note

All claims expressed in this article are solely those of the authors and do not necessarily represent those of their affiliated organizations, or those of the publisher, the editors and the reviewers. Any product that may be evaluated in this article, or claim that may be made by its manufacturer, is not guaranteed or endorsed by the publisher.

## Supplementary Material

Supplementary Figure 1 Evaluation of anti-S1-RBD SARS-CoV-2 Ig-G antibodies through ELISA assays. Results are reported as the optical density value (OD_450nm_) reached after two-fold serial dilutions, starting at 1:200. Samples were obtained before administration of the first dose (pre-immune), two and four weeks after the second dose, and two and four weeks after the disease onset (follow up 1 and 2, respectively). Dotted line indicates the cut-off for the serum dilution at 1:200. **(A–F)** Volunteers 1 to 6 belonging to the 0-14 immunization schedule. **(G–I)** Volunteers 7 to 9 belonging to the 0-28 immunization schedule.

Supplementary Figure 2 Percentage of inhibition of hACE2-spike protein-protein interaction evaluated by a surrogate virus neutralization test (sVNT). Serum samples from nine volunteers were two-fold serially diluted starting to 1:2 and up to 4,096 for neutralizing antibodies detection. Samples were obtained before administration of the first dose (pre-immune), two and four weeks after the second dose, and two and four weeks after the disease onset (follow up 1 and 2, respectively). The dotted line represents the cut-off value at 30% of inhibition **(A–F)** Volunteers 1 to 6 belonging to the 0-14 immunization schedule. **(G–I)** Volunteers 7 to 9 belonging to the 0-28 immunization schedule.

Supplementary Figure 3 T cells responses of breakthrough cases after stimulation with MPs composed of peptides from SARS-CoV-2 proteome. IFN-γ^+^ SFCs of nine breakthrough cases. Data are shown as the fold increase regarding to the pre-immune value for SFCs **(A)** Fold change of IFN-γ^+^ SFCs after stimulation with MPs containing 15-mer peptides from the S protein of SARS-CoV-2. **(B)** Fold change of IFN-γ^+^ SFCs after stimulation with MPs containing 15-mer peptides from the proteome of SARS-CoV-2 excluding the S protein. **(C, D)** Fold change of IFN-γ^+^ SFCs after stimulation with MPs containing 9 to 11-mer peptides from the SARS-CoV-2 proteome.

Supplementary Figure 4 Neutralizing antibody titers of 15 breakthrough cases as compared to 18 vaccinated subjects with no evidence of symptoms associated with COVID-19. Serum samples of individuals were evaluated before vaccine administration (pre-immune), two and four weeks after the second dose. Neutralizing antibodies titers were determined by using **(A)** a surrogate virus neutralizing test and **(B)** a conventional virus neutralizing test. The numbers above the spots indicate the geometric mean titer (GMT) and error bars show the 95% CI of the GMT.

## References

[1] Dong E Du H Gardner L. An Interactive Web-Based Dashboard to Track COVID-19 in Real Time. Lancet Infect Dis (2020) 20:533–4. doi: 10.1016/S1473-3099(20)30120-1 PMC7159018 32087114

[2] Zhu N Zhang D Wang W Li X Yang B Song J. A Novel Coronavirus From Patients With Pneumonia in China, 2019. N Engl J Med (2020) 382:727–33. doi: 10.1056/NEJMoa2001017 PMC7092803 31978945

[3] Dixon BE Wools-Kaloustian KK Fadel WF Duszynski TJ Yiannoutsos C Halverson PK. Symptoms and Symptom Clusters Associated With SARS-CoV-2 Infection in Communitybased Populations: Results From a Statewide Epidemiological Study. PloS One (2021) 16:1–13 . doi: 10.1371/journal.pone.0241875 PMC7990210 33760821

[4] Krammer F. SARS-CoV-2 Vaccines in Development. Nature (2020) 586:516–27. doi: 10.1038/s41586-020-2798-3 32967006

[5] WHO. Covid-19. Draft Landscape of COVID-19 Candidate Vaccines. WHO (2020).

[6] Kyriakidis NC López-Cortés A González EV Grimaldos AB Prado EO. SARS-CoV-2 Vaccines Strategies: A Comprehensive Review of Phase 3 Candidates. NPJ Vaccines (2021) 6:1–17. doi: 10.1038/s41541-021-00292-w 33619260 PMC7900244

[7] Zhang Y Zeng G Pan H Li C Hu Y Chu K. Safety, Tolerability, and Immunogenicity of an Inactivated SARS-CoV-2 Vaccine in Healthy Adults Aged 18-59 Years: A Randomised, Double-Blind, Placebo-Controlled, Phase 1/2 Clinical Trial. Lancet Infect Dis (2021) 21:181–92. doi: 10.1016/S1473-3099(20)30843-4 PMC7832443 33217362

[8] Folegatti PM Ewer KJ Aley PK Angus B Becker S Belij-Rammerstorfer S. Safety and Immunogenicity of the ChAdOx1 Ncov-19 Vaccine Against SARS-CoV-2: A Preliminary Report of a Phase 1/2, Single-Blind, Randomised Controlled Trial. Lancet (2020) 396:467–78. doi: 10.1016/S0140-6736(20)31604-4 PMC7445431 32702298

[9] Mulligan MJ Lyke KE Kitchin N Absalon J Gurtman A Lockhart S. Phase I/II Study of COVID-19 RNA Vaccine BNT162b1 in Adults. Nature (2020) 586:589–93. doi: 10.1038/s41586-020-2639-4 32785213

[10] Polack FP Thomas SJ Kitchin N Absalon J Gurtman A Lockhart S. Safety and Efficacy of the BNT162b2 mRNA Covid-19 Vaccine. N Engl J Med (2020) 383:2603–15. doi: 10.1056/nejmoa2034577 PMC7745181 33301246

[11] Philomina JB Jolly B John N Bhoyar RC Majeed N Senthivel V. Genomic Survey of SARS-CoV-2 Vaccine Breakthrough Infections in Healthcare Workers From Kerala, India. J Infect (2021) 83:237–79. doi: 10.1016/j.jinf.2021.05.018 PMC8143909 34044037

[12] Hacisuleyman E Hale C Saito Y Blachere NE Bergh M Conlon EG. Vaccine Breakthrough Infections With SARS-CoV-2 Variants. N Engl J Med (2021) 384:2212–8. doi: 10.1056/nejmoa2105000 PMC8117968 33882219

[13] Jara A Undurraga EA González C Paredes F Fontecilla T Jara G. Effectiveness of an Inactivated SARS-CoV-2 Vaccine in Chile. N Engl J Med (2021), 385:1–11. doi: 10.1056/NEJMoa2107715 34233097 PMC8279092

[14] Wu Z Hu Y Xu M Chen Z Yang W Jiang Z. Safety, Tolerability, and Immunogenicity of an Inactivated SARS-CoV-2 Vaccine (CoronaVac) in Healthy Adults Aged 60 Years and Older: A Randomised, Double-Blind, Placebo-Controlled, Phase 1/2 Clinical Trial. Lancet Infect Dis (2021) 21:803–12. doi: 10.1016/S1473-3099(20)30987-7 PMC7906628 33548194

[15] Bueno SM Abarca K González PA Gálvez NM Soto JA Duarte LF. Interim Report: Safety and Immunogenicity of an Inactivated Vaccine Against Sars-Cov-2 in Healthy Chilean Adults in A Phase 3 Clinical Trial 2 3 Brief Title: Coronavac03cl Phase 3 Interim Analysis of Safety and Immunogenicity. medRxiv (2021) 2021.03.31.21254494. doi: 10.1101/2021.03.31.21254494

[16] Palacios R Batista AP Albuquerque CSN Patiño EG Santos J do P Tilli Reis Pessoa Conde M. Efficacy and Safety of a COVID-19 Inactivated Vaccine in Healthcare Professionals in Brazil: The PROFISCOV Study. SSRN Electron J (2021). doi: 10.2139/ssrn.3822780

[17] Grifoni A Weiskopf D Ramirez SI Mateus J Dan JM Moderbacher CR. Targets of T Cell Responses to SARS-CoV-2 Coronavirus in Humans With COVID-19 Disease and Unexposed Individuals. Cell (2020) 181:1489–501.e15. doi: 10.1016/j.cell.2020.05.015 32473127 PMC7237901

[18] Valdespino Gómez JL García García MDL. Declaración De Helsinki. Gac Med Mex (2001) 137:387–90. 11859824

[19] WHO. Obesity and Overweight. In: World Heal Organ Media Cent Fact Sheet No 311. WHO (2021).

[20] U. S. Department of Health and Human Services Services USD of H and H Administration F and D Research C for BE. Toxicity Grading Scale for Healthy Adult and Adolescent Volunteers Enrolled in Preventive Vaccine Clinical Trials. Guid Ind (2007).

[21] National Cancer Institute. Common Terminology Criteria for Adverse Events (CTCAE) Version 5.0. NIH Publ (2017). Available at: https://ctep.cancer.gov/protocoldevelopment/electronic_applications/ctc.htm.

[22] WHO Working Group on the Clinical Characterisation and Management of COVID-19 infection. A Minimal Common Outcome Measure Set for COVID-19 Clinical Research. Lancet Infect Dis (2020) 20:e192–7. doi: 10.1016/S1473-3099(20)30483-7 PMC7292605 32539990

[23] Kluge DHHP. Statement – Older People are at Highest Risk From COVID-19, But All Must Act to Prevent Community Spread. World Heal Organ (2020). Available at: https://www.euro.who.int/en/health-topics/health-emergencies/coronavirus-covid-19/statements/statement-older-people-are-at-highest-risk-from-covid-19,-but-all-must-act-to-prevent-community-spread.

[24] Kwetkat A Heppner HJ. Comorbidities in the Elderly and Their Possible Influence on Vaccine Response. Interdiscip Top Gerontol Geriatr (2020) 43:73–85. doi: 10.1159/000504491 32305984

[25] Liu K Chen Y Lin R Han K. Clinical Features of COVID-19 in Elderly Patients: A Comparison With Young and Middle-Aged Patients. J Infect (2020) 80:e14–8. doi: 10.1016/j.jinf.2020.03.005 PMC7102640 32171866

[26] Jin JM Bai P He W Wu F Liu XF Han DM. Gender Differences in Patients With COVID-19: Focus on Severity and Mortality. Front Public Heal (2020) 8:1–6. doi: 10.3389/fpubh.2020.00152 PMC7201103 32411652

[27] Scully EP Haverfield J Ursin RL Tannenbaum C Klein SL. Considering How Biological Sex Impacts Immune Responses and COVID-19 Outcomes. Nat Rev Immunol (2020) 20:442–7. doi: 10.1038/s41577-020-0348-8 PMC7288618 32528136

[28] Kassir R. Risk of COVID-19 for Patients With Obesity. Obes Rev (2020) 21: 1–2. doi: 10.1111/obr.13034 PMC7235532 32281287

[29] Liu M He P Liu HG Wang XJ Li FJ Chen S. Clinical Characteristics of 30 Medical Workers Infected With New Coronavirus Pneumonia. Zhonghua Jie He He Hu Xi Za Zhi (2020) 43:209–14. doi: 10.3760/cma.j.issn.1001-0939.2020.03.014 32164090

[30] Ferrario CM Jessup J Chappell MC Averill DB Brosnihan KB Tallant EA. Effect of Angiotensin-Converting Enzyme Inhibition and Angiotensin II Receptor Blockers on Cardiac Angiotensin-Converting Enzyme 2. Circulation (2005) 111:2605–10. doi: 10.1161/CIRCULATIONAHA.104.510461 15897343

[31] Kow CS Zaidi STR Hasan SS. Cardiovascular Disease and Use of Renin-Angiotensin System Inhibitors in COVID-19. Am J Cardiovasc Drugs (2020) 20:217–21. doi: 10.1007/s40256-020-00406-0 PMC7152511 32281055

[32] Zhang Y Gao Y Qiao L Wang W Chen D. Inflammatory Response Cells During Acute Respiratory Distress Syndrome in Patients With Coronavirus Disease 2019 (COVID-19). Ann Intern Med (2020) 173:402–4. doi: 10.7326/L20-0227 PMC7175423 32282871

[33] Brix TH Hegedüs L Hallas J Lund LC. Risk and Course of SARS-CoV-2 Infection in Patients Treated for Hypothyroidism and Hyperthyroidism. Lancet Diabetes Endocrinol (2021) 9:197–9. doi: 10.1016/S2213-8587(21)00028-0 PMC7906640 33617779

[34] Liao M Liu Y Yuan J Wen Y Xu G Zhao J. Single-Cell Landscape of Bronchoalveolar Immune Cells in Patients With COVID-19. Nat Med (2020) 26:842–4. doi: 10.1038/s41591-020-0901-9 32398875

[35] Su Y Chen D Yuan D Lausted C Choi J Dai CL. Multi-Omics Resolves a Sharp Disease-State Shift Between Mild and Moderate COVID-19. Cell (2020) 183:1479–95.e20. doi: 10.1016/j.cell.2020.10.037 33171100 PMC7598382

[36] Zhang Y Zeng G Pan H Li C Hu Y Chu K. Safety, Tolerability, and Immunogenicity of an Inactivated SARS-CoV-2 Vaccine in Healthy Adults Aged 18–59 Years: A Randomised, Double-Blind, Placebo-Controlled, Phase 1/2 Clinical Trial. Lancet Infect Dis (2021) 21:181–92. doi: 10.1016/S1473-3099(20)30843-4 PMC7832443 33217362

[37] Hariyanto TI Kurniawan A. Thyroid Disease Is Associated With Severe Coronavirus Disease 2019 (COVID-19) Infection. Diabetes Metab Syndr Clin Res Rev (2020) 14:1429–30. doi: 10.1016/j.dsx.2020.07.044 PMC7387272 32755846

[38] Acevedo ML Alonso-Palomares L Bustamante A Gaggero A Paredes F Cortés CP. Infectivity and Immune Escape of the New SARS-CoV-2 Variant of Interest Lambda. medRxiv (2021) 2021.06.28.21259673. doi: 10.1101/2021.06.28.21259673

[39] Bergwerk M Gonen T Lustig Y Amit S Lipsitch M Cohen C. Covid-19 Breakthrough Infections in Vaccinated Health Care Workers. N Engl J Med (2021), 1–11. doi: 10.1056/nejmoa2109072 PMC8362591 34320281

[40] Earle KA Ambrosino DM Fiore-Gartland A Goldblatt D Gilbert PB Siber GR. Evidence for Antibody as a Protective Correlate for COVID-19 Vaccines. Vaccine (2021) 39:4423–8. doi: 10.1016/j.vaccine.2021.05.063 PMC8142841 34210573

[41] Khoury DS Cromer D Reynaldi A Schlub TE Wheatley AK Juno JA. Neutralizing Antibody Levels Are Highly Predictive of Immune Protection From Symptomatic SARS-CoV-2 Infection. Nat Med (2021) 27:1205–11. doi: 10.1038/s41591-021-01377-8 34002089

[42] Estofolete CF Banho CA Campos GRF Marques BC Sacchetto L Ullmann LS. Case Study of Two Post Vaccination SARS-CoV-2 Infections With P1 Variants in Coronavac Vaccinees in Brazil. Viruses (2021) 13:1–10. doi: 10.3390/v13071237 PMC8309964 34206727

[43] Nixon DF Ndhlovu LC. Vaccine Breakthrough Infections With SARS-CoV-2 Variants. N Engl J Med (2021) 385:e7. doi: 10.1056/nejmc2107808 34077640

[44] Strategic Advisory Group of Experts on Immunization - SAGE (WHO). Interim Recommendations for Use of the Inactivated COVID-19 Vaccine, CoronaVac, Developed by Sinovac (2021). Available at: https://apps.who.int/iris/bitstream/handle/10665/341454/WHO-2019-nCoV-vaccines-SAGE-recommendation-Sinovac-CoronaVac-2021.1-eng.pdf.

