# Supplementary figures and images for: Immune Profile and Clinical Outcome of Breakthrough Cases After Vaccination With an Inactivated SARS-CoV-2 Vaccine
Source: Front Immunol. 2021 Sep 29;12:742914. doi: 10.3389/fimmu.2021.742914 (PMC8511644; doi:10.3389/fimmu.2021.742914)

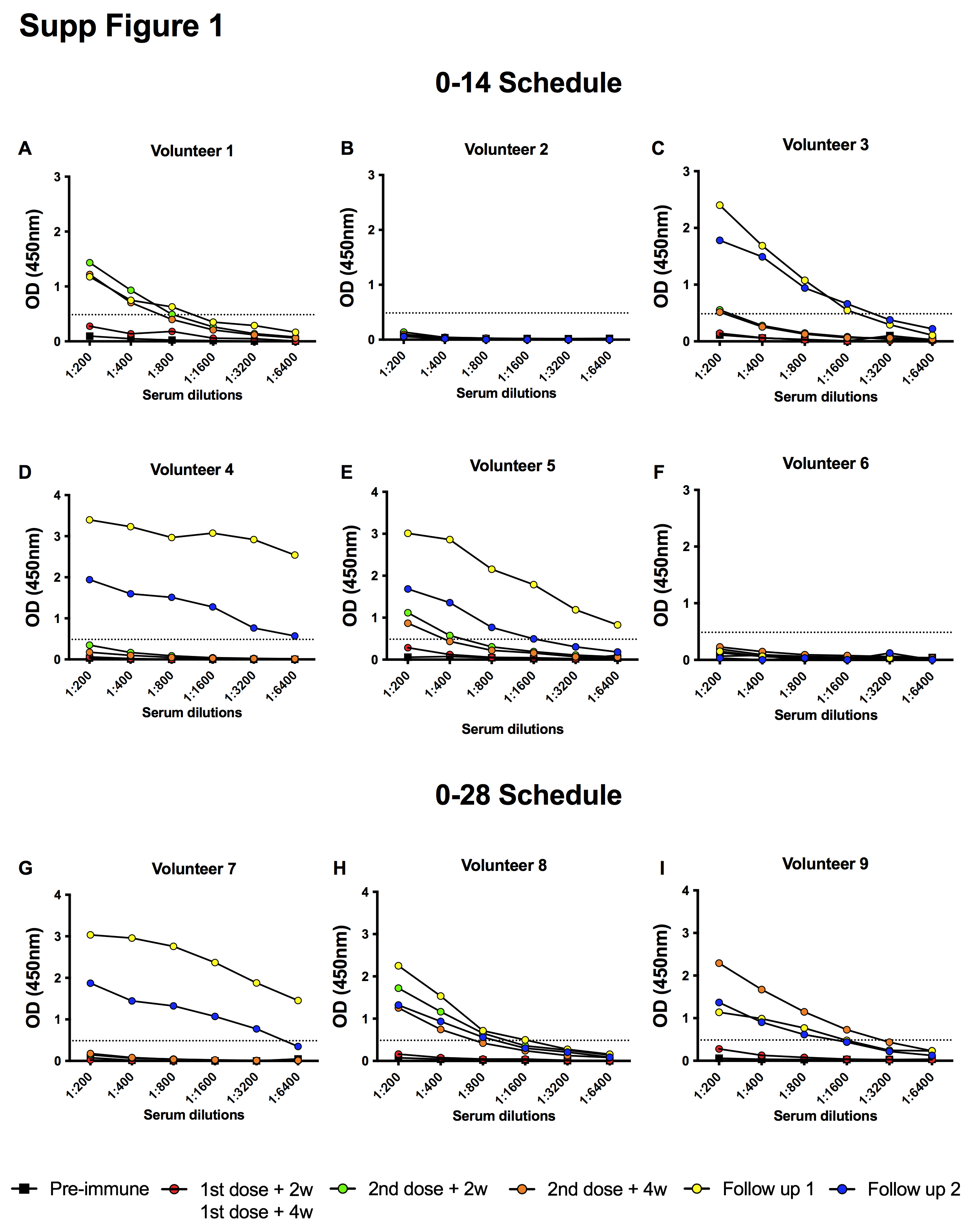

Supplement: Supplementary Figure 1 — Evaluation of anti-S1-RBD SARS-CoV-2 Ig-G antibodies through ELISA assays. Results are reported as the optical density value (OD450nm) reached after two-fold serial dilutions, starting at 1:200. Samples were obtained before administration of the first dose (pre-immune), two and four weeks after the second dose, and two and four weeks after the disease onset (follow up 1 and 2, respectively). Dotted line indicates the cut-off for the serum dilution at 1:200. (A–F) Volunteers 1 to 6 belonging to the 0-14 immunization schedule. (G–I) Volunteers 7 to 9 belonging to the 0-28 immunization schedule. [file Image_1.tiff]

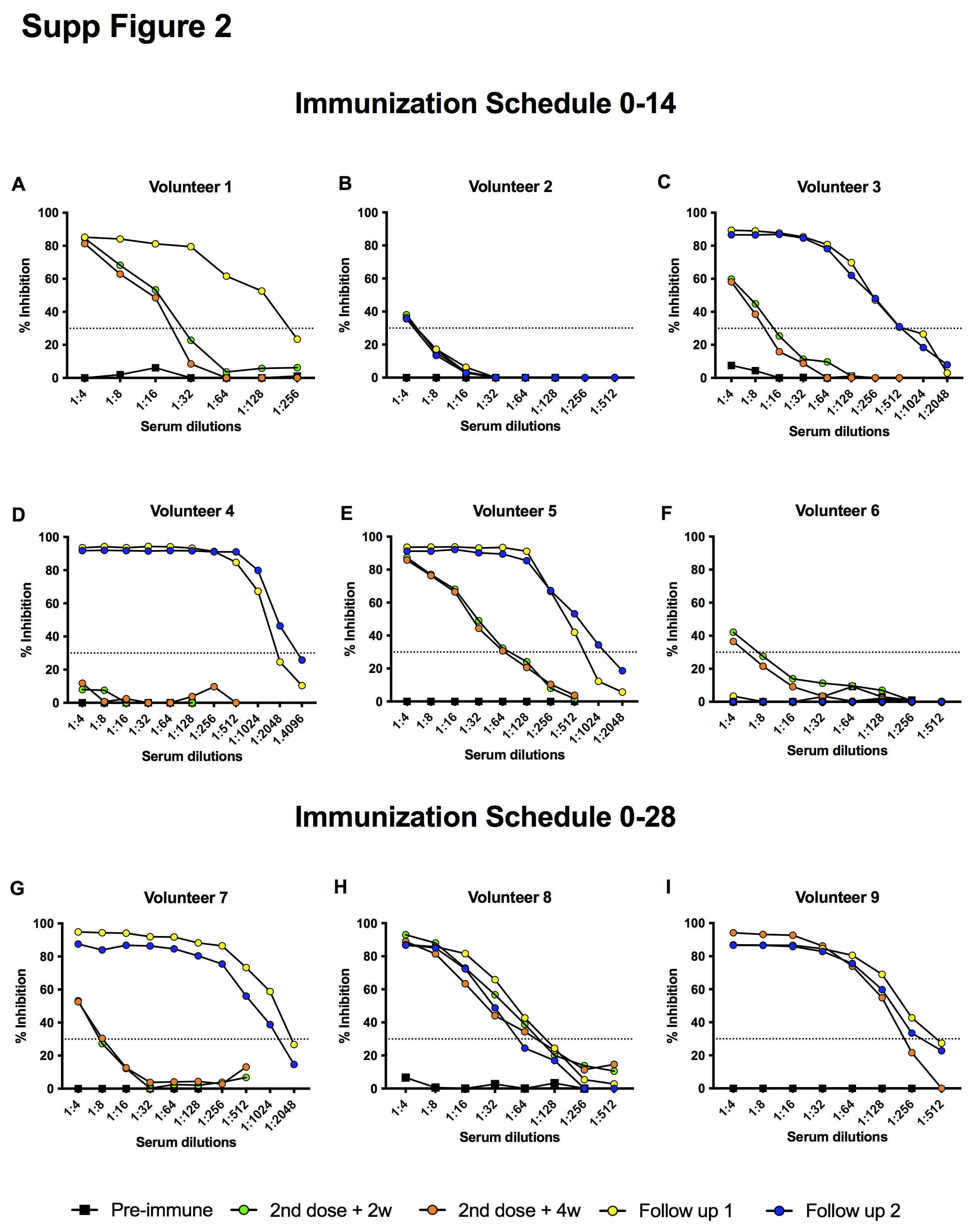

Supplement: Supplementary Figure 2 — Percentage of inhibition of hACE2-spike protein-protein interaction evaluated by a surrogate virus neutralization test (sVNT). Serum samples from nine volunteers were two-fold serially diluted starting to 1:2 and up to 4,096 for neutralizing antibodies detection. Samples were obtained before administration of the first dose (pre-immune), two and four weeks after the second dose, and two and four weeks after the disease onset (follow up 1 and 2, respectively). The dotted line represents the cut-off value at 30% of inhibition (A–F) Volunteers 1 to 6 belonging to the 0-14 immunization schedule. (G–I) Volunteers 7 to 9 belonging to the 0-28 immunization schedule. [file Image_2.tiff]

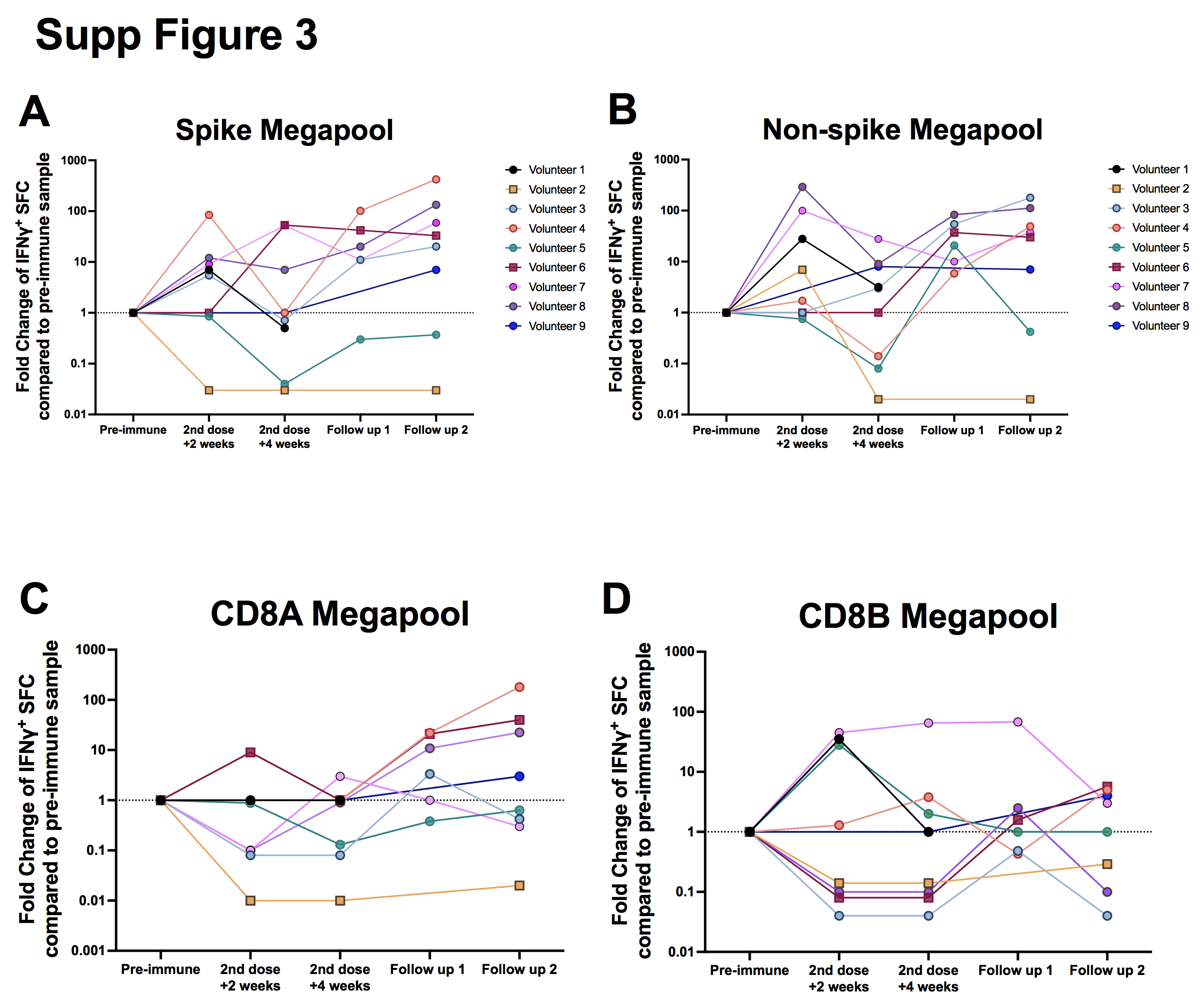

Supplement: Supplementary Figure 3 — T cells responses of breakthrough cases after stimulation with MPs composed of peptides from SARS-CoV-2 proteome. IFN-γ+ SFCs of nine breakthrough cases. Data are shown as the fold increase regarding to the pre-immune value for SFCs (A) Fold change of IFN-γ+ SFCs after stimulation with MPs containing 15-mer peptides from the S protein of SARS-CoV-2. (B) Fold change of IFN-γ+ SFCs after stimulation with MPs containing 15-mer peptides from the proteome of SARS-CoV-2 excluding the S protein. (C, D) Fold change of IFN-γ+ SFCs after stimulation with MPs containing 9 to 11-mer peptides from the SARS-CoV-2 proteome. [file Image_3.tiff]

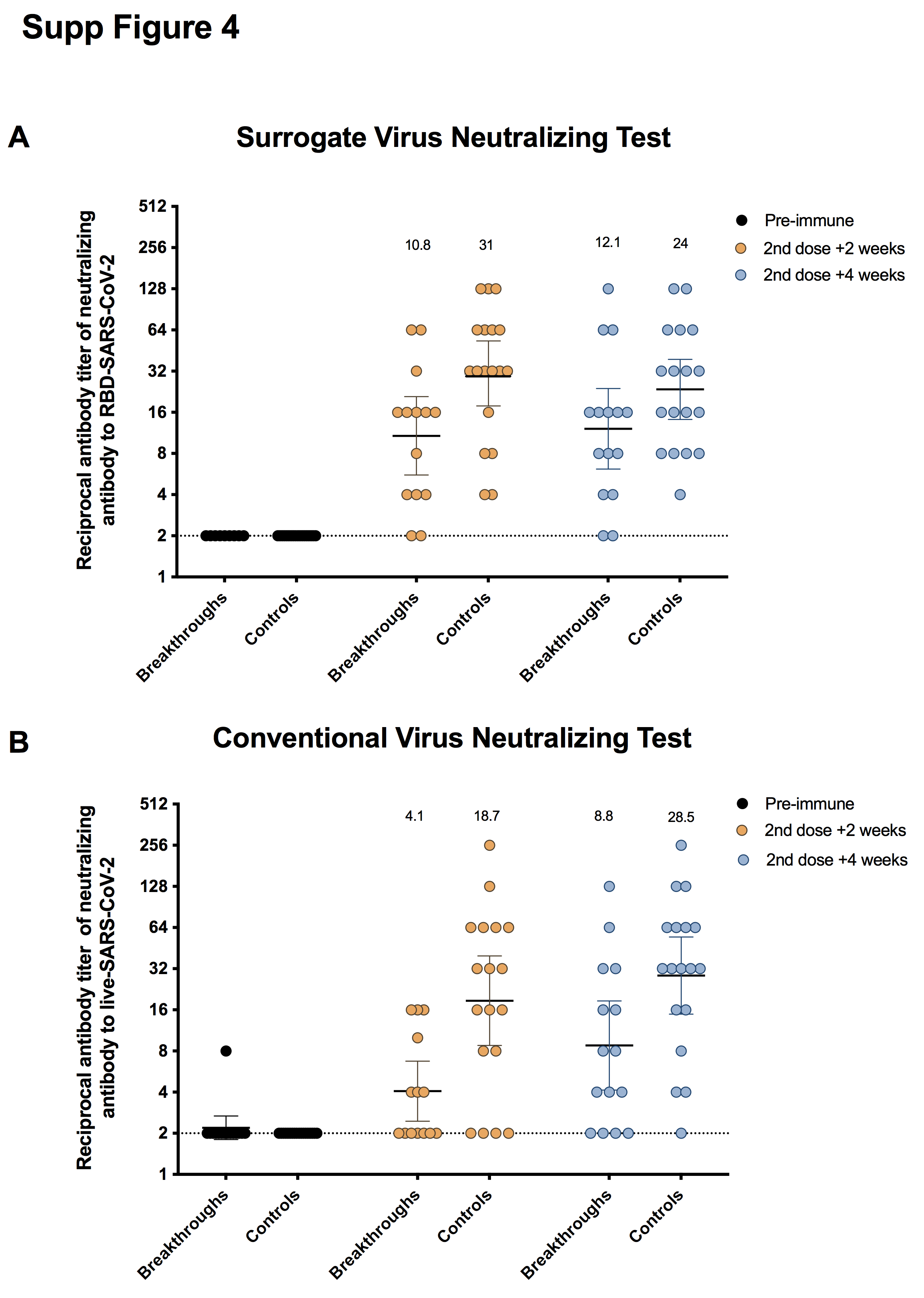

Supplement: Supplementary Figure 4 — Neutralizing antibody titers of 15 breakthrough cases as compared to 18 vaccinated subjects with no evidence of symptoms associated with COVID-19. Serum samples of individuals were evaluated before vaccine administration (pre-immune), two and four weeks after the second dose. Neutralizing antibodies titers were determined by using (A) a surrogate virus neutralizing test and (B) a conventional virus neutralizing test. The numbers above the spots indicate the geometric mean titer (GMT) and error bars show the 95% CI of the GMT. [file Image_4.tiff]
