# Supplementary material for: Immune Profile and Clinical Outcome of Breakthrough Cases After Vaccination With an Inactivated SARS-CoV-2 Vaccine
Source: Front Immunol. 2021 Sep 29;12:742914. doi: 10.3389/fimmu.2021.742914 (PMC8511644; doi:10.3389/fimmu.2021.742914)
Supplement: Supplementary file 5 [file Presentation_1.pdf]

Supplementary Appendix

1. CoronaVac03CL Study Team / Sept 14, 2021

<u>Center CL1</u>: Áreas Ambulatorias Marcoleta - Pontificia Universidad Católica de Chile.

1. Alvaro Miguel Rojas Gonzalez,
2. Maria Soledad Navarrete Bello,
3. Constanza Belen Del Rio Solis,
4. Dinely Valeska Del Pino Lavin,
5. Natalia Elizabeth Aguirre Concha,
6. Franco Vega Farias,
7. Acsa Raquel Salgado Ovalle,
8. Thomas Quinteros,
9. Alma Muñoz,
10. Patricio Astudillo,
11. Monique Nicole Le Corre.

<u>Center CL2</u>: Clínica San Carlos de Apoquindo - Red de Salud UC-Christus

1. Marcela Potin Santander
2. Sofía Aljaro Ehrenberg
3. Sófia López Coloma
4. Tania Weil Valjalo
5. Gema Pérez Alarcón
6. Melan Peralta Kong
7. Consuelo Zamanillo Moreira

<u>Center CL4</u>: Clínica Los Andes - Universidad de Los Andes

1. Paula Guzmán Merino,
2. Francisca Aguirre Boza,
3. Aarón Cortés Rojas,
4. Luis Federico Bátiz,
5. Javiera Francisca Pérez Velásquez,
6. Karen Pamela Apablaza García,
7. Lorena Yates Barsotti,
8. María de los Ángeles Valdés Valdés,
9. Bernardita Hurtado,
10. Veronique Venteneul,
11. Constanza Astorga.
12. Maria Francisca Bossans
13. Ximena Correa
14. Pilar Navarro
15. Javiera Lagas

<u>Center CL5</u>: Clínica Alemana - Universidad del Desarrollo

1. Paula Andrea Muñoz-Venturelli,

2. Pablo Agustín Vial,
3. Andrea Ingrid Schilling Redlich,
4. Daniela Pavez Azurmendi,
5. Inia Andrea Pérez Villa,
6. Amy Lisa Riviotta,
7. Francisca Gonzalez Mc Cowley,
8. Francisca Pilar Urrutia Goldsack,
9. Alejandra Isabel Del Río Weldt,
10. Claudia Andrea del Carmen Asenjo Lobos,
11. Bárbara Paulina Vargas Latorre,
12. Francisca Valentina Castro Fuentes,
13. Alejandra Patricia Acuña Rogel,
14. Javiera Constanza Gúzman Cancino,
15. Camila Alejandra Astudillo Griffiths.
16. Camila Portilla Fuentes
17. Paulina Bustos Alarcón,
18. Carlos Delfino Garay

<u>Center CL6</u>: Hospital Clínico Félix Bulnes - Universidad San Sebastián

1. Carlos M Pérez,
2. Pilar Espinoza,
3. Andrea Martínez,
4. Marcela Arancibia,
5. Harold Romero,
6. Cecilia Bustamante,
7. María Loreto Pérez,
8. Natalia Uribe,
9. Viviana Silva,
10. Bernardita Morice,
11. Marco Pérez,
12. Clara Alvarado.

<u>Center CL7</u>: Hospital Dr. Gustavo Fricke - Universidad de Valparaíso

1. Marcela González,
2. Nataly Martínez,
3. Camila Molina,
4. Juliette Sánchez.

<u>Center CL8</u>: Hospital Carlos Van Buren- Universidad de Valparaíso

1. Daniela Fuentes Hulse,
2. Yolanda Calvo Toro,
3. Mariela Cepeda Corrales,
4. Rosario Lemus Manzur,
5. Constance Marucich Baeza,
6. Cecilia Cornejo Beas

<u>Center CL9</u>: Complejo Asistencial Dr. Sótero del Rio

1. Paulina Donato Inostroza,
2. Martin Lasso Barreto,
3. María Iturrieta Meléndez,
4. María Acuña Schlegel,
5. Ada Cascone Scarpati,
6. Raymundo Rojas Araya,
7. Camila Sepúlveda Contreras,
8. Mario Alex Contreras,
9. Yessica Campisto Sanhueza,
10. Pablo González Sanhueza,
11. Zoila Quizhpi Mejias,
12. Mariella Lopez García,
13. Vania Pizzeghello Salfate,
14. Stephannie Silva Monsalve.

2. Members of the Independent Data Safety Monitoring Committee.

Luis Delpiano, MD, Pediatric Infectologist, Hospital San Borja Arriarán, Santiago, Chile.

Macarena Lagos, MD, Immunologist, Clínica Las Condes and Hospital Padre Hurtado, Santiago, Chile.

Gloria Icaza, MD, Epidemiologist and Statitician, Universidad de Talca, Talca, Chile.

Leonardo Chanqueo, MD, Adult Infectologist, Hospital San Juan de Dios, Santiago, Chile.

Mónica Imarai, PhD, Universidad de Santiago, Santiago, Chile.
