# Supplementary material for: Immune Profile and Clinical Outcome of Breakthrough Cases After Vaccination With an Inactivated SARS-CoV-2 Vaccine
Source: Front Immunol. 2021 Sep 29;12:742914. doi: 10.3389/fimmu.2021.742914 (PMC8511644; doi:10.3389/fimmu.2021.742914)
Supplement: Supplementary file 6 [file Table_1.docx]

**Supplementary Table 1 (Related to Figure 1, 2 and 3). Data shows the reciprocal of antibody titers of S1 IgG, reciprocal antibody titer of neutralizing antibodies to RBD-SARS-CoV-2 (sVNT) or live SARS-CoV 2 (cVNT) and fold change of IFN-γ^+^ spots forming cells (SFC) after stimulations with SARS-CoV-2 MegaPools.**

| Volunteer | Parameter | Pre-immune | 1st dose + 2 or 4 weeks* | 2nd dose +2 weeks | 2nd dose + 4 weeks | Follow up 1 | Follow up 2 |
| --- | --- | --- | --- | --- | --- | --- | --- |
| 1 | anti-S1 IgG antibodies | 100 | 1600 | 6400 | 6400 | 6400 | ·· |
|  | Neutralizing antibodies (sVNT) | 2 | ·· | 16 | 16 | 128 | ·· |
|  | Neutralizing antibodies (cVNT) | 2 | - | 10 | 4 | 256 | ·· |
|  | Fold change of IFN-g+ SFCs (15-mer) | 1 | ·· | 24·5 | 2·8 | ·· | ·· |
|  | Fold change of IFN-g+ SFCs (9 to 11-mer) | 1 | ·· | 142 | 2 | ·· | ·· |
| 2 | anti-S1 IgG antibodies | 100 | 100 | 100 | 100 | 100 | 100 |
|  | Neutralizing antibodies (sVNT) | 2 | ·· | 4 | 4 | 4 | 4 |
|  | Neutralizing antibodies (cVNT) | 2 | ·· | 2 | 2 | 16 | 32 |
|  | Fold change of IFN-g+ SFCs (15-mer) | 1 | ·· | 3·8 | 0·02 | ·· | 0·02 |
|  | Fold change of IFN-g+ SFCs (9 to 11-mer) | 1 | ·· | 0·03 | 0·03 | ·· | 0·05 |
| 3 | anti-S1 IgG antibodies | 100 | 100 | 1200 | 3200 | 6400 | 6400 |
|  | Neutralizing antibodies (sVNT) | 2 | ·· | 8 | 8 | 512 | 512 |
|  | Neutralizing antibodies (cVNT) | 8 | ·· | 16 | 16 | 128 | 512 |
|  | Fold change of IFN-g+ SFCs (15-mer) | 1 | ·· | 4·9 | 1·0 | 16·4 | 40·0 |
|  | Fold change of IFN-g+ SFCs (9 to 11-mer) | 1 | ·· | 0·06 | 0·06 | 1.5 | 0·17 |
| 4 | anti-S1 IgG antibodies | 100 | 100 | 100 | 100 | 6400 | 6400 |
|  | Neutralizing antibodies (sVNT) | 2 | ·· | 2 | 2 | 1024 | 2048 |
|  | Neutralizing antibodies (cVNT) | 2 | ·· | 2 | 2 | 512 | 512 |
|  | Fold change of IFN-g+ SFCs (15-mer) | 1 | ·· | 12·0 | 0.3 | 17·8 | 95·5 |
|  | Fold change of IFN-g+ SFCs (9 to 11-mer) | 1 | ·· | 1·3 | 3·6 | 1·9 | 16·7 |
| 5 | anti-S1 IgG antibodies | 100 | 800 | 6400 | 6400 | 6400 | 6400 |
|  | Neutralizing antibodies (sVNT) | 2 | ·· | 64 | 64 | 512 | 1024 |
|  | Neutralizing antibodies (cVNT) | 2 | ·· | 4 | 8 | 256 | 512 |
|  | Fold change of IFN-g+ SFCs (15-mer) | 1 | ·· | 0·82 | 0·05 | 6·56 | 0·38 |
|  | Fold change of IFN-g+ SFCs (9 to 11-mer) | 1 | ·· | 3·9 | 0·3 | 0·4 | 0·67 |
| 6 | anti-S1 IgG antibodies | 100 | 100 | 100 | 100 | 100 | 100 |
|  | Neutralizing antibodies (sVNT) | 2 | ·· | 4 | 4 | 2 | 2 |
|  | Neutralizing antibodies (cVNT) | 2 | ·· | 2 | 2 | 2 | 8 |
|  | Fold change of IFN-g+ SFCs (15-mer) | 1 | ·· | 1 | 27 | 39·5 | 31·5 |
|  | Fold change of IFN-g+ SFCs (9 to 11-mer) | 1 | ·· | 0·8 | 0·2 | 3·1 | 8·3 |
| 7 | anti-S1 IgG antibodies | 100 | 100 | 100 | 200 | 6400 | 6400 |
|  | Neutralizing antibodies (sVNT) | 2 | ·· | 4 | 8 | 1024 | 1024 |
|  | Neutralizing antibodies (cVNT) | 2 | ·· | 2 | 8 | 256 | 256 |
|  | Fold change of IFN-g+ SFCs (15-mer) | 1 | ·· | 54·3 | 38·3 | 10·5 | 48·3 |
|  | Fold change of IFN-g+ SFCs (9 to 11-mer) | 1 | ·· | 1·2 | 4·4 | 2·5 | 0·3 |
| 8 | anti-S1 IgG antibodies | 100 | 100 | 200 | 1600 | 6400 | 1600 |
|  | Neutralizing antibodies (sVNT) | 2 | ·· | 64 | 64 | 64 | 32 |
|  | Neutralizing antibodies (cVNT) | 2 | ·· | 16 | 4 | 128 | 128 |
|  | Fold change of IFN-g+ SFCs (15-mer) | 1 | ·· | 152 | 8 | 51·5 | 122·5 |
|  | Fold change of IFN-g+ SFCs (9 to 11-mer) | 1 | ·· | 0·08 | 0·68 | 8·2 | 15·32 |
| 9 | anti-S1 IgG antibodies | 100 | 800 |  | 6400 | 6400 | 6400 |
|  | Neutralizing antibodies (sVNT) | 2 | ·· | ·· | 128 | 256 | 256 |
|  | Neutralizing antibodies (cVNT) | 2 | ·· | ·· | 32 | 256 | 128 |
|  | Fold change of IFN-g+ SFCs (15-mer) | 1 | ·· | ·· | 4·5 | ·· | 7 |
|  | Fold change of IFN-g+ SFCs (9 to 11-mer) | 1 | ·· | ·· | 1 | · | 3·5 |
