# Supplementary material for: Immune Profile and Clinical Outcome of Breakthrough Cases After Vaccination With an Inactivated SARS-CoV-2 Vaccine
Source: Front Immunol. 2021 Sep 29;12:742914. doi: 10.3389/fimmu.2021.742914 (PMC8511644; doi:10.3389/fimmu.2021.742914)
Supplement: Supplementary file 7 [file Table_2.docx]

**Supplementary table 2 (Related to figure 3). Spots forming cells after stimulation with SARS-CoV-2 MegaPools***

|  | Stimuli | Pre-immune | 2nd dose +2 weeks | 2nd dose + 4 weeks | Follow up 1 | Follow up 2 |
| --- | --- | --- | --- | --- | --- | --- |
| 1 | Spike | 2 | 14 | 1 | ·· | ·· |
|  | Non-Spike | 11 | 305 | 35 | ·· | ·· |
|  | S + Non-S | 13 | 319 | 36 | ·· | ·· |
|  | CD8-A | 8 | 1 | 1 | ·· | ·· |
|  | CD8-B | 4 | 141 | 1 | ·· | ·· |
|  | CD8-A +CD8-B | 1 | 142 | 2 | ·· | ·· |
| 2 | Spike | 39 | 1 | 1 | ·· | 1 |
|  | Non-Spike | 49 | 332 | 1 | ·· | 1 |
|  | S + Non-S | 88 | 333 | 2 | ·· | 2 |
|  | CD8-A | 78 | 1 | 1 | ·· | 2 |
|  | CD8-B | 1 | 1 | 1 | ·· | 2 |
|  | CD8-A +CD8-B | 79 | 2 | 2 | ·· | 4 |
| 3 | Spike | 7 | 38 | 5 | 77 | 141 |
|  | Non-Spike | 1 | 1 | 3 | 54 | 179 |
|  | S + Non-S | 8 | 39 | 8 | 131 | 320 |
|  | CD8-A | 12 | 1 | 1 | 40 | 5 |
|  | CD8-B | 23 | 1 | 1 | 11 | 1 |
|  | CD8-A +CD8-B | 35 | 2 | 2 | 51 | 6 |
| 4 | Spike | 1 | 84 | 1 | 101 | 421 |
|  | Non-Spike | 7 | 12 | 1 | 41 | 343 |
|  | S + Non-S | 8 | 96 | 2 | 142 | 764 |
|  | CD8-A | 1 | 1 | 1 | 22 | 180 |
|  | CD8-B | 14 | 18 | 53 | 6 | 70 |
|  | CD8-A +CD8-B | 15 | 19 | 54 | 28 | 250 |
| 5 | Spike | 27 | 23 | 1 | 8 | 10 |
|  | Non-Spike | 12 | 9 | 1 | 248 | 5 |
|  | S + Non-S | 39 | 32 | 2 | 256 | 15 |
|  | CD8-A | 8 | 7 | 1 | 3 | 5 |
|  | CD8-B | 1 | 28 | 2 | 1 | 1 |
|  | CD8-A +CD8-B | 9 | 35 | 3 | 4 | 6 |
| 6 | Spike | 1 | 1 | 53 | 42 | 33 |
|  | Non-Spike | 1 | 1 | 1 | 37 | 30 |
|  | S + Non-S | 2 | 2 | 54 | 79 | 63 |
|  | CD8-A | 1 | 9 | 1 | 21 | 40 |
|  | CD8-B | 12 | 1 | 1 | 19 | 68 |
|  | CD8-A +CD8-B | 13 | 10 | 2 | 40 | 108 |
| 7 | Spike | 3 | 27 | 157 | 33 | 177 |
|  | Non-Spike | 3 | 299 | 83 | 30 | 113 |
|  | S + Non-S | 6 | 326 | 230 | 63 | 290 |
|  | CD8-A | 42 | 5 | 126 | 40 | 11 |
|  | CD8-B | 1 | 45 | 65 | 68 | 3 |
|  | CD8-A +CD8-B | 43 | 50 | 191 | 108 | 14 |
| 8 | Spike | 1 | 12 | 7 | 20 | 133 |
|  | Non-Spike | 1 | 292 | 9 | 83 | 112 |
|  | S + Non-S | 2 | 304 | 16 | 103 | 245 |
|  | CD8-A | 17 | 1 | 16 | 185 | 382 |
|  | CD8-B | 8 | 1 | 1 | 20 | 1 |
|  | CD8-A +CD8-B | 25 | 2 | 17 | 205 | 383 |
| 9 | Spike | 1 | ·· | 1 | ·· | 7 |
|  | Non-Spike | 1 | ·· | 8 | ·· | 7 |
|  | S + Non-S | 2 | ·· | 9 | ·· | 14 |
|  | CD8-A | 1 | ·· | 1 | ·· | 3 |
|  | CD8-B | 1 | ·· | 1 | ·· | 4 |
|  | CD8-A +CD8-B | 2 | ·· | 2 | ·· | 7 |

* Data normalized by subtraction of DMSO background
