# Supplementary material for: Immune Profile and Clinical Outcome of Breakthrough Cases After Vaccination With an Inactivated SARS-CoV-2 Vaccine
Source: Front Immunol. 2021 Sep 29;12:742914. doi: 10.3389/fimmu.2021.742914 (PMC8511644; doi:10.3389/fimmu.2021.742914)
Supplement: Supplementary file 8 [file Table_3.docx]

|  |  |  |  |  | sVNT | | | cVNT | | | Fold change 15-mer MP (ELISPOT) | | | Fold change 9 to 11-mer MP (ELISPOT) | | |
| --- | --- | --- | --- | --- | --- | --- | --- | --- | --- | --- | --- | --- | --- | --- | --- | --- |
| Volunteer | Biological Sex | Age | Immunization Schedule | Date of Immunization  (m/d/y) | Pre-immune | 2nd dose +2 weeks | 2nd dose +4 weeks | Pre-immune | 2nd dose + 2 weeks | 2nd dose +4 weeks | Pre-immune | 2nd dose + 2 weeks | 2nd dose +4 weeks | Pre-immune | 2nd dose + 2 weeks | 2nd dose +4 weeks |
| 1 | F | 18-59 | 0-14 | 12/3/20 | 2 | 4 | 8 | 2 | 2 | 16 | 1 | 27·00 | 11·50 | 1 | 2·33 | 4·00 |
| 2 | F | 18-59 | 0-14 | 12/9/20 | 2 | 32 | 32 | 2 | 256 | 128 | 1 | 1·44 | 1·35 | 1 | 2·78 | 0·97 |
| 3 | F | 18-59 | 0-14 | 12/10/20 | 2 | 64 | 64 | 2 | 32 | 64 | 1 | 0·53 | 3·53 | 1 | 0·67 | 0·67 |
| 4 | M | 18-59 | 0-14 | 12/10/20 | 2 | 32 | 32 | 2 | 128 | 256 | 1 | 2·70 | 15·80 | 1 | 0·27 | 8·00 |
| 5 | M | 18-59 | 0-14 | 12/10/20 | 2 | 32 | 16 | 2 | 64 | 64 | 1 | 0·13 | 0·69 | 1 | 0·20 | 0·20 |
| 6 | F | 18-59 | 0-14 | 12/10/20 | 2 | 32 | 16 | 2 | 64 | 128 | 1 | 7·71 | 2·29 | 1 | 4·00 | 0·67 |
| 7 | F | 18-59 | 0-14 | 12/10/20 | 2 | 32 | 16 | 2 | 64 | 64 | 1 | 0·40 | 1·40 | 1 | 0·33 | 0·67 |
| 8 | F | 18-59 | 0-14 | 12/11/20 | 2 | 4 | 4 | 2 | 2 | 32 | 1 | 0·20 | 5·80 | 1 | 0·67 | 18·33 |
| 9 | F | 18-59 | 0-14 | 12/11/20 | 2 | 16 | 8 | 2 | 16 | 64 | 1 | 14·50 | 1·50 | 1 | 1·80 | 0·93 |
| 10 | F | 18-59 | 0-14 | 12/14/20 | 2 | 32 | 32 | 2 | 8 | 8 | 1 | 0·50 | 2·75 | 1 | 1·64 | 3·18 |
| 11 | M | >60 | 0-14 | 12/16/20 | 2 | 128 | 64 | 2 | 8 | 32 | 1 | 0·50 | 17·75 | 1 | 0·40 | 0·40 |
| 12 | M | >60 | 0-14 | 12/21/20 | 2 | 64 | 128 | 2 | 32 | 32 | 1 | 1·00 | 47·50 | 1 | 53·00 | 36·50 |
| 13 | M | >60 | 0-28 | 2/2/21 | 2 | 8 | 8 | 2 | 2 | 2 | 1 | 0·50 | 0·50 | 1 | 0·22 | 0·44 |
| 14 | M | >60 | 0-28 | 2/3/21 | 2 | 8 | 8 | 2 | 2 | 4 | 1 | 14·67 | 15·67 | 1 | 6·00 | 8·50 |
| 15 | F | 18-59 | 0-28 | 1/27/21 | 2 | 128 | 128 | 2 | 32 | 16 | 1 | 1·00 | 19·00 | 1 | 1·00 | 8·00 |
| 16 | F | 18-59 | 0-28 | 1/29/21 | 2 | 64 | 32 | 2 | 64 | 32 | 1 | 15·67 | 9·67 | 1 | 1·00 | 11·00 |
| 17 | F | 18-59 | 0-28 | 1/29/21 | 2 | 64 | 16 | 2 | 16 | 32 | 1 | 0·67 | 0·33 | 1 | 1·00 | 14·50 |
| 18 | F | 18-59 | 0-28 | 2/1/21 | 2 | 128 | 64 | 2 | 16 | 4 | 1 | 8·67 | 13·00 | 1 | 0·32 | 0·08 |

**Supplementary Table 3 (Related to Figure 5). Reciprocal antibody titers of S1 IgG, reciprocal antibody titer of neutralizing antibodies to RBD-SARS-CoV-2 (sVNT) or live SARS-CoV 2 (cVNT) and fold change of IFN-γ^+^ spots forming cells (SFC) after stimulations with SARS-CoV-2 MegaPools for the control cohort.**

*Gray shading: Female. No shading: Male
